# Hypoxia-mediated promotion of glucose metabolism in non-small cell lung cancer correlates with activation of the EZH2/FBXL7/PFKFB4 axis

**DOI:** 10.1038/s41419-023-05795-z

**Published:** 2023-05-13

**Authors:** Jing Zhou, Yang Lin, Xiuhua Kang, Zhicheng Liu, Juntao Zou, Fei Xu

**Affiliations:** Department of Respiratory and Critical Care Medicine, the First Affiliated Hospital of Nanchang University, Nanchang, 330006 China

**Keywords:** Non-small-cell lung cancer, Non-small-cell lung cancer

## Abstract

F-box/LRR-repeat protein 7 (FBXL7) was predicted as a differentially expressed E3 ubiquitin ligase in non-small cell lung cancer (NSCLC), which has been suggested to influence cancer growth and metastasis. In this study, we aimed to decipher the function of FBXL7 in NSCLC and delineate the upstream and downstream mechanisms. FBXL7 expression was verified in NSCLC cell lines and GEPIA-based tissue samples, after which the upstream transcription factor of FBXL7 was bioinformatically identified. The substrate PFKFB4 of the FBXL7 was screened out by tandem affinity purification coupled with mass-spectrometry (TAP/MS). FBXL7 was downregulated in NSCLC cell lines and tissue samples. FBXL7 ubiquitinated and degraded PFKFB4, thus suppressing glucose metabolism and malignant phenotypes of NSCLC cells. Hypoxia-induced HIF-1α upregulation elevated EZH2 and inhibited FBXL7 transcription and reduced its expression, thus promoting PFKFB4 protein stability. By this mechanism, glucose metabolism and the malignant phenotype were enhanced. In addition, knockdown of EZH2 impeded tumor growth through the FBXL7/PFKFB4 axis. In conclusion, our work reveals that the EZH2/FBXL7/PFKFB4 axis plays a regulatory role in glucose metabolism and tumor growth of NSCLC, which is expected to be potential biomarkers for NSCLC.

## Introduction

Lung cancer is one of the most prevalent cancers and the leading causes of cancer-related mortality in males across the globe [1, 2]. Non-small cell lung cancer (NSCLC) is the most common form of lung cancer [3], accounting for 85% of all lung cancers [4]. Despite advancements in the treatment of NSCLC [5], the overall survival rates [6] for NSCLC are still unfavorable, especially in metastatic stage [3]. Thus, extensive investigations into the disease biology and mechanisms of tumor metastasis [7] are required to advance early detection and multimodal care.

It is becoming clear that hypoxia is associated with unsatisfactory prognosis in NSCLC, which points to the significance of targeting hypoxia to improve NSCLC outcomes [8]. Recent evidence suggests that enhancer of zeste homolog 2 (EZH2) is substantially expressed in tumor cells exposed to hypoxia [9, 10]. Whereas, EZH2 knockdown can delay the progression of NSCLC in vivo by enhancing anti-tumor immune responses [11]. In addition, the effect of EZH2 in malignancy has been documented to be largely associated with glucose metabolism of cancer cells, which is recognized as a hallmark of cancer growth and metastasis [12, 13]. Moreover, a strong interaction between EZH2 and SKP1-CUL1-F-box (SCF) E3 ubiquitin ligases in malignant diseases has been reported in multiple studies [14, 15].

Of note, our microarray data analysis had identified the only differentially expressed E3 ubiquitin ligase F-box/LRR-repeat protein 7 (FBXL7) related to NSCLC. FBXL7, a component of the SCF E3 ubiquitin ligases essential for maintaining the tissue homeostasis in response to normal and stress conditions, especially in malignant diseases [16, 17]. Overexpression of FBXL7 results in inhibition of invasive properties of pancreatic cancer cells [18]. FBXL7 may be also a useful biomarker for the prediction of complete pathologic response in patients with ovarian cancer [19]. The biological role of FBXL7 in NSCLC was the focus of the current study. 6-phosphofructo-2-kinase/fructose-2,6-biphosphatase 4 (PFKFB4) has been identified to enhance the chemoresistance in small-cell lung cancer (SCLC) and is an indicative of poor therapeutic response and prognosis in this disease [20]. In addition, PFKFB4 can control metabolic flux through allosteric regulation of glycolysis; evidence has suggested that restriction of PFKFB4 may confer beneficial effects for cancer treatment as its ectopic expression exhibits a supporting role in the anabolic metabolism in p53-deficient cancer cells [21]. These reports imply the possible participation of hypoxia in the glucose metabolism and tumor growth of NSCLC involving the interaction with EZH2, FBXL7, and PFKFB4.

## Materials and methods

### Ethics statement

The current study was approved by the Animal Ethics Committee of the First Affiliated Hospital of Nanchang University and performed according to the Guide for the Care and Use of Laboratory Animals published by the US National Institutes of Health.

### Database retrieval

NSCLC-related gene expression datasets GSE12472, GSE27262, GSE101929, and GSE118370 were retrieved from the GEO database. GSE12472 contains 28 normal samples and 35 NSCLC samples, GSE27262 contains 25 normal samples and 25 NSCLC samples, GSE101929 contains 34 normal samples and 32 NSCLC samples, and GSE118370 contains 6 normal samples and 6 NSCLC samples. R “limma” package was used to screen the differentially expressed genes (DEGs) with |log fold change (FC)| > 1, *p* < 0.05 as the threshold. A total of 501 E3 ubiquitin ligases were obtained from iUUCD database. Gene expression in NSCLC was analyzed using GEPIA and UALCAN databases. Kaplan-Meier Plotter database was applied for correlation analysis of genes with the prognosis of NSCLC. UCSC database was used to predict the transcriptional regulators of gene promoter binding.

### Experimental protocols in vitro

Seven human NSCLC cell lines (A549, H3122, H460, HCC827, H1299, H1650, and H1975), human normal lung epithelial cell line (BEAS-2B) and the 293T cells were all purchased from American Type Culture Collection (ATCC, Manassas, VA). All cells were tested for mycoplasma contamination before the experiments.

Cell culture under normoxic conditions: cells were cultured in RPMI 1640 medium containing 10% FBS, 2 mM glutamine, and 1% penicillin-streptomycin in a 21% O_2_ incubator at 37 °C. The medium was renewed every three days. Cells were detached with 0.25% trypsin/EDTA at 37 °C for 1 min and then sub-cultured, upon reaching 80% confluence.

Cell culture under hypoxic conditions: the medium was renewed, upon reaching 70–80% confluence. NSCLC cells were cultured with 1% O_2_ at 37 °C for 2, 4, 7, or 10 h for maintenance of culture under normoxic conditions (21% O_2_) as controls.

Cells were transduced with oe-NC, oe-FBXL7, HA-FBXL7, Myc-PFKFB4, vector (Vec), alone or combined with cycloheximide (CHX; 20 μg/mL, Beyotime Biotechnology Co., Ltd., Shanghai, China) for 0, 3, 6, 9, and 12 h, respectively, or si-NC, si-EZH2, oe-NC, oe-FBXL7, oe-FBXL7 + oe-PFKFB4, and oe-FBXL7 + oe-PFKFB4 + 2-deoxy-D-glucose (2-DG).

Cells under normoxic or hypoxic conditions were treated with si-NC and si-EZH2. Cells under hypoxic conditions were treated with si-EZH2 + si-FBXL7 or combined with 2-DG (16 mM, 154-17-6, MCE). Lipofectamine 3000 reagent (Invitrogen, Carlsbad, CA) or Lipofectamine 2000 reagent (Invitrogen) was applied for cell treatment. 48 h after the transduction, the cells were cultured in medium containing puromycin (1 g/ml, A1113803, Thermo Fisher) to screen for stably transduced cell line. The overexpression or knockdown efficiency was confirmed by RT-qPCR or Western blot.

### RNA isolation and quantitation

TRIzol-extracted RNA was reverse transcribed into cDNA using the PrimeScript™ RT-qPCR Kit (Takara, Mountain View, CA). RT-qPCR was conducted using SYBR Premix Ex Taq™ (Takara) on LightCycler 480 system (Roche Diagnostics, Pleasanton, CA). As normalized to Actin, fold changes were evaluated using the 2^−ΔΔCt^ method [22]. Primers were provided by Shanghai General Biotechnology Co., Ltd. (Shanghai, China), with sequences listed in Supplementary Table 1.

### Protein extraction and Western blot analysis

Total proteins from cells were prepared with enhanced RIPA lysis buffer with protease inhibitor, with the concentration determined by a BCA kit (Boster). Lysates were loaded on SDS-PAGE and electro-transferred onto PVDF membranes. After being blocked using 5% BSA, membranes underwent overnight incubation at 4 °C with the diluted primary antibodies against EZH2 (5246, 1:1000, Cell Signaling Technologies [CST], Beverly, MA), FBXL7 (sc-374319, 1:1000, Santa Cruz Biotechnology, Santa Cruz, CA), HA (ab9110, 1:1000, Abcam, Cambridge, UK), PFKFB4 (ab137785, 1:1000, Abcam), HIF-1α (36169, 1:1000, CST), Myc (ab32, 1:1000, Abcam) and Actin (ab8226, 1:2000, Abcam). The next day, the membrane was incubated with HRP-labeled secondary antibody goat anti-mouse (ab6808, 1:2000, Abcam) or goat anti-rabbit (ab6721, 1:2000, Abcam) at room temperature for 1 h. An ECL reagent (EMD Millipore, Billerica, MA) was used for image developing and band intensity was calculated using Image J software, with β-actin as the internal reference.

### Cell viability, migration, invasion, and apoptosis assays

CCK-8 kits (K1018, Apexbio) were used to examine cell viability [23]. The plated cells (5 × 10^3^ cells/well) were cultured for 1, 2, and 3 days and incubated with 10 µL of CCK-8 solution at 37 °C for 2 h, followed by OD value (at 450 nm) measurement.

Transwell inserts (pore size of 8 μm; Corning Incorporated, Corning, NY) uncoated (migration) or coated (invasion) with Matrigel reagent (BD Bioscience, San Diego, CA) were used for cell migration and invasion measurement [24]. After being stained with 0.1% crystal violet, cells were photographed under an inverted microscope (Carl Zeiss, Jena, Germany) and counted using ImageJ software.

Apoptosis was evaluated on the basis of the instructions of FITC-Annexin V cell apoptosis detection kit (4830-01-K, R&D Systems) [25], where Annexin-V-FITC and PI were mixed at a ratio of 1:2 to prepare Annexin-V-FITC/PI staining solution.

### Tandem affinity purification coupled with mass-spectrometry (TAP/MS)

FBXL7 was cloned into pcDNA3.1 vector and 3× Flag and HA purification tag were fused to its N-terminus. Flag-HA-FBXL7 plasmid was transduced into 293T cells using Lipofectamine 2000 reagent. A total of about 4 × 10^8^ cells were collected and lysed with RIPA lysis buffer, whereupon Flag-HA-FBXL7 and the interacting protein complex were separated and purified with Flag-HA dual labeling purification kit (TP0010-5RXN, Sigma-Aldrich Chemical Company, St Louis, MO). Anti-Flag agarose beads (MA1-91878-BTIN, Sigma-Aldrich) was used to enrich Flag-HA-FBXL7, and 3×Flag peptide was used to elute Flag-HA-FBXL7 and its interacting proteins, followed by another enrichment with anti-HA agarose beads (S190138, Sigma-Aldrich). The magnetic beads containing Flag-HA-FBXL7 and its interacting protein complex were washed, added with 1×SDS loading buffer, heated, and treated with SDS-PAGE. LS-MS was performed to identify all proteins in the complex.

### Co-immunoprecipitation (Co-IP)

Cells were lysed with protease inhibitor- and phosphatase inhibitor-containing EBC buffer (50 mM Tris, pH 7.5, 120 mM NaCl, and 0.5% NP-40). A 5 μL aliquot of lysate was used as input, while the remaining underwent incubation with 10 μL Protein G magnetic beads at 4 °C for 1 h. Next, the cell lysate was incubated overnight at 4 °C with 20 µL of anti-HA agarose beads (S190138, Sigma-Aldrich) or anti-Flag agarose beads (MA1-91878-BTIN, Sigma-Aldrich), Myc (ab32, 1:000, Abcam), and PFKFB4 (ab137785, 1:1000, Abcam). The recovered immunocomplexes were washed with NETN buffer, followed by Western blot analysis.

### Measurement of glucose metabolites

Glucose uptake assay kit (K676-100, Biovision, Mountain View, CA) was used for glucose uptake evaluation [26]. The cells were starved for glucose by pre-incubating with Krebs-Ringer-Phosphate-HEPES (KRPH) buffer containing 2% BSA, followed by incubation with 10 mM 2-DG for 20 min. The cells were lysed, then frozen/thawed, and heated at 85 °C for 40 min. Cell lysates were neutralized and centrifuged to harvest the supernatant for glucose uptake assessment at 412 nm in a microplate reader.

For pyruvate assay, cell lysates lysed in 4 volume of the pyruvate assay buffer (Biovision) were assayed by pyruvate colorimetric assay kit (K609, Biovision), followed by measurement at 570 nm using a microplate reader.

ATP assay kit (700410, Cayman Chemical, Ann Arbor, MI) was used for ATP content assay. ATP sample buffer-lysed cell lysates were incubated with a mixture containing D-Luciferin and Luciferase at room temperature for 20 min, with fluorescence examined by SpectraMax i3x (Molecular Devices).

Pyruvate colorimetric assay kit (K676-100, Biovision) was applied for measurement of lactate production. The supernatant of cells was collected for measurement of lactate production, which was examined at 450 nm with a microplate reader.

In terms of lactate production in tumor tissues of nude mice, tumor tissues were homogenized in Assay Buffer (Biovision). After centrifugation, the soluble fraction was then examined and normalized to protein concentration. For ATP level analysis, cells (5 × 10^5^/mL) were collected and resuspended in 100 μL of ATP analysis buffer (Biovision). After centrifugation, the supernatant was harvested for ATP determination at 570 nm using a microplate reader.

### Extracellular acid ratio (ECAR) detection

The Seahorse extracellular Flux Analyzer XF96 (Seahorse Bioscience) and Seahorse XF glycolysis stress test kit (103020-100, Agilent, Beijing, China) were used to monitor in vitro cell metabolic alternations [27]. For detection of the real-time glycolytic rate, as reflected by ECAR (mpH/min), cells underwent incubation with 10 mM glucose, 1 µM oligomycin, and 80 mM 2-deoxyglucose.

### Reactive oxygen species (ROS) accumulation

The plated cells were incubated with PBS containing DCFH-DA (10 µM, used to measure the total ROS in cells) or Mitosox (5 µM, used to measure the ROS in mitochondria) for 45 min or 10 min. Fluorescence at 485/535 nm and 510/580 nm was examined using a fluorescence microplate.

### Luciferase activity assay

To detect the binding of HIF-1α to EZH2, the EZH2 regulatory sequence (ccgCGTGcctg) was inserted into the pGL3 promoter vector (WT-EZH2), and MUT-EZH2 was then constructed using site-directed mutagenesis kit (Takara), with CGTG mutated to GCAC. Transfection of constructed vectors into cells was conducted using Lipofectamine 2000 reagent (Thermo Fisher), and cell culture was carried out under normoxia (21% O_2_) and hypoxia (1% O_2_).

The binding of HIF-1α to the promoter region FBXL7 was determined in the same way. Specifically, the FBXL7 regulatory sequence was inserted into the pGL3 promoter vector (WT-FBXL7), and MUT-FBXL7 was then constructed using site-directed mutagenesis kit. The constructed vectors were co-transduced with si-NC or si-EZH2 into cells using Lipofectamine 2000 reagent (Thermo Fisher) for 48 h. Firefly luciferase activity detection was conducted using the Dual-Luciferase Reporter Assay System (Promega), normalized to renilla luciferase activity.

### Chromatin immunoprecipitation (ChIP)

EZ-Magna ChIP kit (EMD Millipore) was applied for ChIP assay [28]. DNA-protein cross-linking was produced by paraformaldehyde (4%) fixing, followed by ultrasonic treatment to produce chromatin fragments (200–300 bp). Next, the antibody-coated magnetic protein A beads were used to immunoprecipitate the lysates, followed by RT-qPCR for precipitated DNA. The antibodies (Abcam) used included IgG (rabbit, ab171870, serving as NC), EZH2 (ab191250, 1:50) and HIF-1α (ab228649, 1:50).

### GST pull-down assay

FBXL7 cDNA was isolated by RT-qPCR, and cloned into pGEX-4T-1 vector with GST tag by T4 DNA ligase. PFKFB4 cDNA was cloned into the XhoI and EcoRI sites of the pET-28a vector with His tag. GST-FBXL7 and His-PFKFB4 recombinant plasmids were transformed into *E. coli* BL21 (DE3) respectively. GST-FBXL7 or His-PFKFB4 recombinant protein was synthesized. Next, purified GST or GST-tagged FBXL7 proteins were mixed with glutathione-Sepharose beads (GE Healthcare) for 1 h at 4 °C and then with 1 µg His-PFKFB4 for 1 h at 4 °C. The proteins were then incubated with the purified His-PFKFB4 overnight at 4 °C. The collected beads were subjected to western blot analysis [29], with such antibodies as anti-GST (Beijing TransGen Biotech Co., Ltd., Beijing, China) and anti-His (CWBio, Beijing, China).

### Experimental protocols in vivo

Male BALB/c nude mice (4-week-old, Hunan SJA Laboratory Animal Co., Ltd., Hunan, China) were enrolled in this study. Cells treated with sh-NC + oe-NC, sh-EZH2 + oe-NC, and sh-EZH2 + oe-PFKFB4 were subcutaneously inoculated into the armpit of these mice at a density of 1 × 10^7^/200 μL to establish a xenograft model of NSCLC. Tumor size was measured every 3 days.

### Histological, immunohistochemical, and TUNEL staining

Tumor tissues of nude mice were fixed with 4% paraformaldehyde, paraffin-embedded, and cut into sections at a thickness of 4 μm. Paraffin sections were dewaxed in xylene, hydrated with gradient alcohol, and then stained with hematoxylin and eosin (HE) [30].

Sections were blocked with goat serum after antigen retrieval. The immunohistochemical staining included primary antibodies against FBXL7 (sc-374319, 1:100, Santa Cruz), Ki-67 (ab15580, 1:100, Abcam), EZH2 (5246, 1:100, CST), and PFKFB4 (ab137785, 1:100, Abcam). The next day, the sections were re-probed with the goat anti-rabbit IgG (ab6721, 1:100, Abcam) and goat anti-nude mouse IgG (ab205719, 1:100, Abcam). After DAB development and hematoxylin counterstaining, image was acquired using a Nikon ECLIPSE Ti (Japan) microscope system [31].

TUNEL staining kit (Shanghai Beyotime) was used to assess cell apoptosis in the tumor tissues [32]. Samples were incubated with biotin labeling solution at 37 °C for 60 min in the dark, followed by incubation in Streptavidin-HRP working solution. Thereafter, the samples were subjected to DAB development and DAPI counterstaining before observation under a confocal microscope (Olympus, Tokyo, Japan). Quantification of cell apoptosis was conducted using Image Pro-Plus 6.0 software.

### Statistical analysis

All data from three independent experiments were described as mean ± standard deviation and analyzed using GraphPad Prism 8.0 (GraphPad Software, La Jolla, CA). Data between two groups were compared using independent sample *t*-test. Variables among multiple groups were compared using one-way analysis of variance (ANOVA) with Tukey’s post hoc test. Tumor volume at various time points was compared by Bonferroni-corrected repeated measures ANOVA. The difference was statistically significant at *p* < 0.05.

## Results

### FBXL7 is poorly expressed in NSCLC samples and cells

**Fig. 1:**
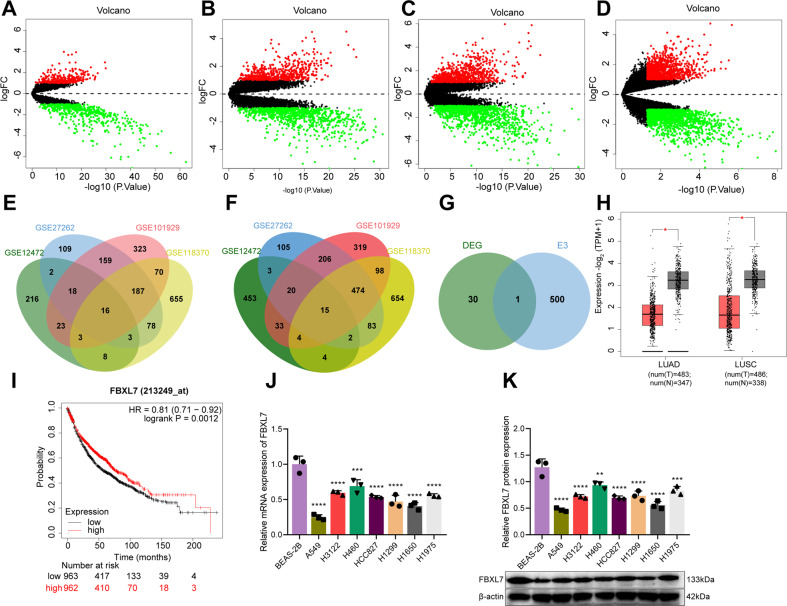
FBXL7 is poorly expressed in NSCLC samples and cells. **A** A volcano plot of DEGs in 28 normal samples and 35 NSCLC samples in the GSE12472 dataset. Red indicates upregulated DEGs, green indicates downregulated DEGs, and black indicates no difference in gene expression. **B** A volcano plot of DEGs in 25 normal samples and 25 NSCLC samples in the GSE27262 dataset. Red indicates upregulated DEGs, green indicates downregulated DEGs, and black indicates no difference in gene expression. **C** A volcano plot of DEGs in 34 normal samples and 32 NSCLC samples in the GSE101929 dataset. Red indicates upregulated DEGs, green indicates downregulated DEGs, and black indicates no difference in gene expression. **D** A volcano plot of DEGs in 6 normal samples and 6 NSCLC samples in the GSE118370 dataset. Red indicates upregulated DEGs, green indicates downregulated DEGs, and black indicates no difference in gene expression. **E** Venn diagram of the 16 upregulated DEGs from the four datasets. **F** Venn diagram of the 15 downregulated DEGs from the four datasets. **G** Venn diagram showing the intersection (FBXL7) of the DEGs from the four datasets and 501 E3 ubiquitin ligases from the iUUCD database. **H** Expression of FBXL7 mRNA was downregulated in NSCLC tissue samples, as analyzed using GEPIA. Red box: tumor samples, gray box: normal samples; LUSC: lung squamous cell carcinoma, LUAD: lung adenocarcinoma. **I** Kaplan-Meier analysis showing the positive correlation between FBXL7 expression and the overall survival rate of patients. Red: high expression of FBXL7; black: low expression of FBXL7. **J** Expression of FBXL7 was downregulated in NSCLC cells (as compared with normal lung epithelial cells), as determined by RT-qPCR. **K** Western blot analysis showing the downregulated FBXL7 protein in NSCLC cells (as compared with normal lung epithelial cells). **p < 0.01, ***p < 0.001, ****p < 0.0001, compared with BEAS-2B cells. The cell experiment was repeated three times independently.

Differential analysis of the NSCLC-related datasets GSE12472, GSE27262, GSE101929, and GSE118370 yielded 16 upregulated genes and 15 downregulated genes in NSCLC samples (Fig. 1A–F). Among them, FBXL7 is the only differentially expressed E3 ubiquitin ligase (Fig. 1G). In addition, analysis using GEPIA database suggested that the expression of FBXL7 was downregulated in NSCLC samples (Fig. 1H). Kaplan–Meier analysis showed that FBXL7 expression was positively correlated with the overall survival of patients with NSCLC (Fig. 1I). Therefore, we speculated FBXL7 as a key tumor suppressor gene in NSCLC. As compared with normal human lung epithelial cells, lower expression of FBXL7 was noted in human NSCLC cell lines (A549, H3122, H460, HCC827, H1299, H1650, H1975) (Fig. 1J, K). The A549 and H1650 cell lines, where the lowest expression was witnessed, were selected for subsequent experiments. These results suggested that FBXL7 was poorly expressed in NSCLC cells and may be related to the malignant progression of NSCLC.

### Forced expression of FBXL7 inhibits the malignant phenotypes of NSCLC cells and induces their apoptosis

**Fig. 2:**
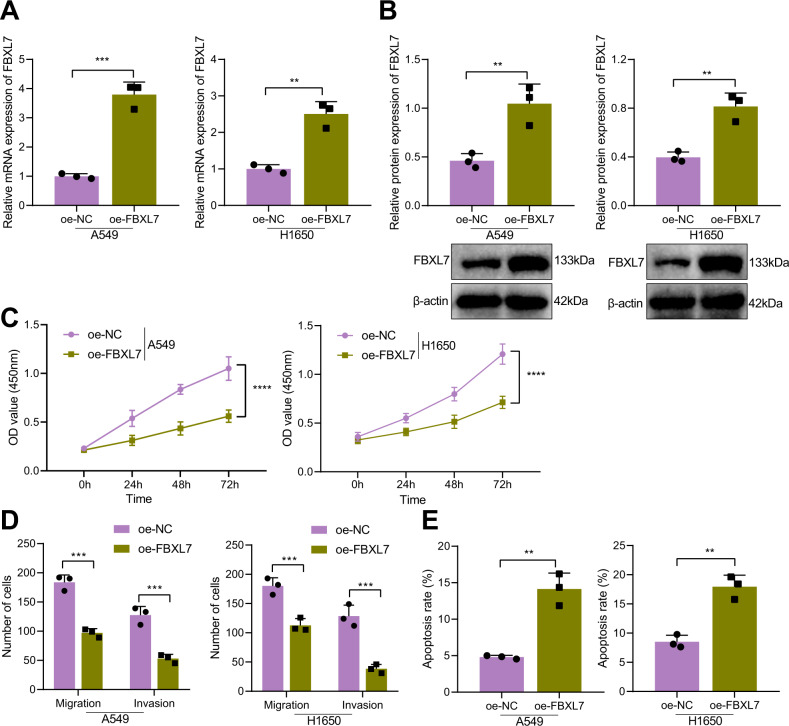
FBXL7 arrests the malignant characteristics of NSCLC cells. **A** Expression of FBXL7 mRNA was elevated in A549 and H1650 cells transduced with oe-FBXL7, as determined by RT-qPCR. **B** Western blot analysis showing the elevated FBXL7 protein expression in A549 and H1650 cells transduced with oe-FBXL7. **C** Viability of A549 and H1650 cells was inhibited in response to oe-FBXL7, as analyzed by CCK-8 assay. **D** Migration and invasion of A549 and H1650 cells were inhibited in response to oe-FBXL7 analyzed by Transwell assays. **E** Apoptosis of A549 and H1650 cells was promoted in response to oe-FBXL7, as analyzed by flow cytometry. **p < 0.01, ***p < 0.001, ****p < 0.0001. The cell experiment was repeated three times independently.

Next, we conducted cell experiments to explore the effect of FBXL7 on the growth of NSCLC cells. The results of RT-qPCR and Western blot analysis showed increased expression of FBXL7 mRNA and protein in A549 and H1650 cells transduced with oe-FBXL7 (Fig. 2A, B). CCK-8 and Transwell assay results indicated that overexpression of FBXL7 led to significant inhibition of viability, migration, and invasion of A549 and H1650 cells (Fig. 2C, D). In addition, the results of flow cytometry revealed that the apoptosis of A549 and H1650 cells overexpressing FBXL7 was stimulated (Fig. 2E). The above data indicate that overexpression of FBXL7 can suppress the malignant phenotypes of NSCLC cells while facilitating their apoptosis.

### FBXL7 promotes the ubiquitination-mediated degradation of PFKFB4

**Fig. 3:**
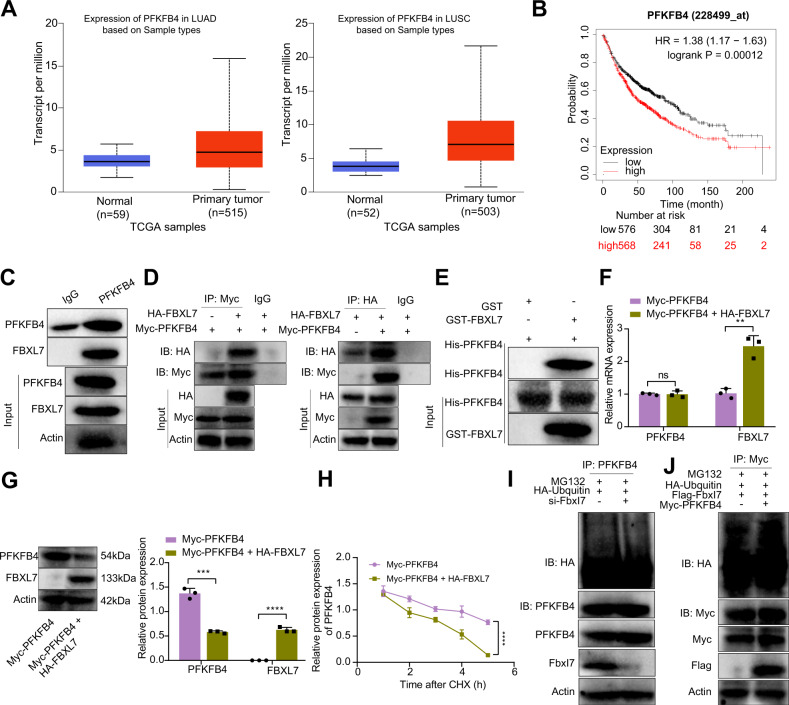
FBXL7 enhances the ubiquitination-mediated degradation of PFKFB4. **A** PFKFB4 expression was elevated in NSCLC tissue samples, as analyzed by UALCAN database. LUSC: lung squamous cell carcinoma; LUAD: lung adenocarcinoma. **B** Kaplan–Meier analysis showing the negative correlation between PFKFB4 and the overall survival rate of NSCLC patients. Red: high expression of PFKFB4; black: low expression of PFKFB4. **C** Co-IP analysis of the interaction of endogenous FBXL7 with PFKFB4 in 293T cells. **D** Co-IP analysis of the interaction of exogenous FBXL7 with PFKFB4 in 293T cells. **E** GST-pull down assay analysis of the direct interaction between FBXL7 and PFKFB4 in 293T cells. **F** PFKFB4 mRNA expression in A549 cells wasn’t changed in response to oe-FBXL7, as determined by RT-qPCR. **G** Western blot analysis showing inhibited PFKFB4 protein expression in A549 cells in response to oe-FBXL7. **H** Western blot analysis showing enhanced degradation of PFKFB4 protein in 293T cells in response to oe-FBXL7 and CHX. **I** Ubiquitination level of PFKFB4 protein was reduced in 293T cells in the presence of si-FBXL7, as determined by IP assay. **J** Ubiquitination level of PFKFB4 protein was elevated in 293T cells in the presence of oe-FBXL7, as determined by IP assay. **p < 0.01, ***p < 0.001, ****p < 0.0001. ns *p* > 0.05. The cell experiment was repeated three times independently.

In order to clarify the molecular mechanism of FBXL7 inhibiting the malignant progression of NSCLC cells, we used TAP-MS to identify the substrate of FBXL7 ubiquitin ligase (Supplementary Table 2). Analysis using UALCAN database indicated that the expression of PFKFB4 was significantly upregulated in NSCLC samples (Fig. 3A), and it was negatively correlated with the overall survival time of NSCLC patients (Fig. 3B). Therefore, we speculated that FBXL7 may mediate the glycolytic pathway through ubiquitination, which in turn affects NSCLC progression.

Besides, the results of Co-IP assay showed that endogenous PFKFB4 interacted with FBXL7 (Fig. 3C). In addition, we transferred HA-labeled FBXL7 and Myc-labeled PFKFB4 into 293T cells, and used HA- or Myc-labeled antibody for immunoprecipitation. Co-IP results demonstrated that exogenous PFKFB4 also interacted with FBXL7 (Fig. 3D). To verify the direct interaction between FBXL7 and PFKFB4, GST-tag was used to label FBXL7 and His to label PFKFB4. GST pull-down assay data exhibited the interaction of His-PFKFB4 with GST-FBXL7 (Fig. 3E). Meanwhile, the results of RT-qPCR and Western blot analysis showed that transduction with oe-FBXL7 in A549 cells reduced the protein expression of PFKFB4 without affecting its mRNA expression (Fig. 3F, G). This result suggested that PFKFB4 may be a substrate of FBXL7, which may degrade PFKFB4 protein through ubiquitination.

CHX was applied for detection of the effect of FBXL7 on the half-life of PFKFB4 protein. Western blot analysis results explained faster degradation of PFKFB4 protein in 293T cells with oe-FBXL7 (Fig. 3H). In addition, IP assay results presented a decline in the ubiquitination level of PFKFB4 protein in the absence of FBXL7, while an opposite result was observed following FBXL7 overexpression (Fig. 3I, J).

These data supported that PFKFB4 was highly expressed in NSCLC tissue samples and overexpression of FBXL7 can ubiquitinate and degrade PFKFB4 protein.

### FBXL7 reduces the expression of PFKFB4, thereby inhibiting glucose metabolism and malignant phenotype of NSCLC cells

The aforementioned results allowed us to validate whether PFKFB4 was involved in the inhibiting effect of FBXL7 on the malignant characteristics of NSCLC cells.

**Fig. 4:**
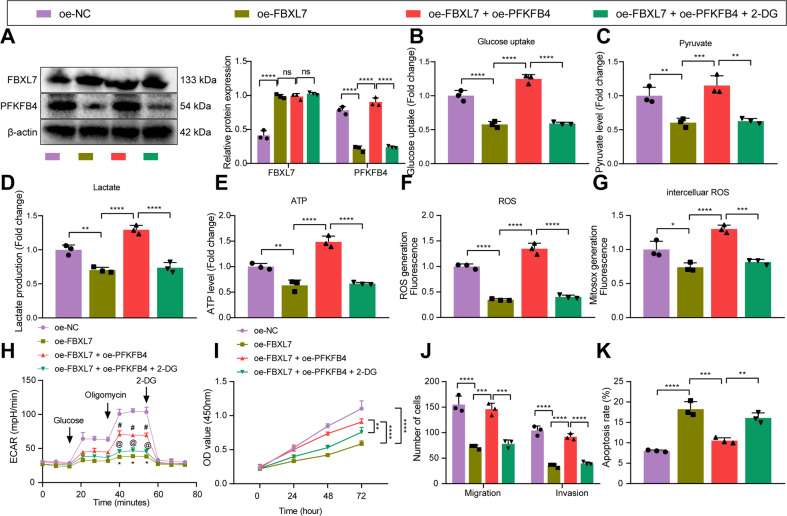
FBXL7 downregulates PFKFB4 to inhibit glucose metabolism and malignant phenotypes of NSCLC cells. **A** Western blot analysis of FBXL7 and PFKFB4 proteins in A549 cells in response to oe-FBXL7, oe-FBXL7 + oe-PFKFB4, or oe-FBXL7 + oe-PFKFB4 + 2-DG. **B** Glucose uptake measurement in response to oe-FBXL7, oe-FBXL7 + oe-PFKFB4, or oe-FBXL7 + oe-PFKFB4 + 2-DG. **C** Pyruvate level measurement in response to oe-FBXL7, oe-FBXL7 + oe-PFKFB4, or oe-FBXL7 + oe-PFKFB4 + 2-DG. **D** Lactate production measurement in response to oe-FBXL7, oe-FBXL7 + oe-PFKFB4, or oe-FBXL7 + oe-PFKFB4 + 2-DG. **E** ATP level measurement in response to oe-FBXL7, oe-FBXL7 + oe-PFKFB4, or oe-FBXL7 + oe-PFKFB4 + 2-DG. **F** ROS content measurement in A549 cells in response to oe-FBXL7, oe-FBXL7 + oe-PFKFB4, or oe-FBXL7 + oe-PFKFB4 + 2-DG. **G** ROS content measurement in mitochondrion in response to oe-FBXL7, oe-FBXL7 + oe-PFKFB4, or oe-FBXL7 + oe-PFKFB4 + 2-DG. **H** Glycolysis of A549 cells in response to oe-FBXL7, oe-FBXL7 + oe-PFKFB4, or oe-FBXL7 + oe-PFKFB4 + 2-DG as detected by ECAR assay (^#^*p* < 0.05 vs. the oe-FBXL7 group; ^@^*p* < 0.05 vs. the oe-FBXL7 + oe-PFKFB4 group). **I** Viability of A549 cells in response to oe-FBXL7, oe-FBXL7 + oe-PFKFB4, or oe-FBXL7 + oe-PFKFB4 + 2-DG, as analyzed by CCK-8 assay. **J** Migration and invasion of A549 cells in response to oe-FBXL7, oe-FBXL7 + oe-PFKFB4, or oe-FBXL7 + oe-PFKFB4 + 2-DG, as analyzed by Transwell assay. **K** Apoptosis of A549 cells in response to oe-FBXL7, oe-FBXL7 + oe-PFKFB4, or oe-FBXL7 + oe-PFKFB4 + 2-DG, as analyzed by flow cytometry. *p < 0.05, **p < 0.01, ***p < 0.001, ****p < 0.0001. ns *p* > 0.05. The cell experiment was repeated three times independently.

Western blot analysis results illustrated an increase in FBXL7 expression yet a reduction in PFKFB4 expression in A549 cells upon transduction with oe-FBXL7. Simultaneous overexpression of FBXL7 and PFKFB4 failed to alter FBXL7 expression while elevating PFKFB4 expression. Further treatment with 2-DG decreased PFKFB4 expression without altering FBXL7 expression (Fig. 4A).

As shown in Fig. 4B–G, oe-FBXL7 reduced glucose uptake, pyruvate level, ATP level, lactate production, and ROS content while oe-FBXL7 + oe-PFKFB4 led to opposite results. Further treatment with 2-DG caused the same results as those of oe-FBXL7.

In Figure 4H, oe-FBXL7 was noted to weaken the glycolysis, which was restored upon oe-PFKFB4. In the presence of oe-FBXL7 + oe-PFKFB4 + 2-DG, glycolysis was attenuated. In addition, cell viability, migration, and invasion were repressed while cell apoptosis was augmented following oe-FBXL7, the effect of which was reversed by oe-PFKFB4. Further treatment with 2-DG resulted in the consistent results with those of oe-FBXL7 (Fig. 4I–K).

Collectively, these results indicated that overexpression of FBXL7 can inhibit glucose metabolism by downregulating PFKFB4, thereby inhibiting the malignant phenotype of NSCLC cells.

### EZH2 inhibits the transcription of FBXL7

**Fig. 5:**
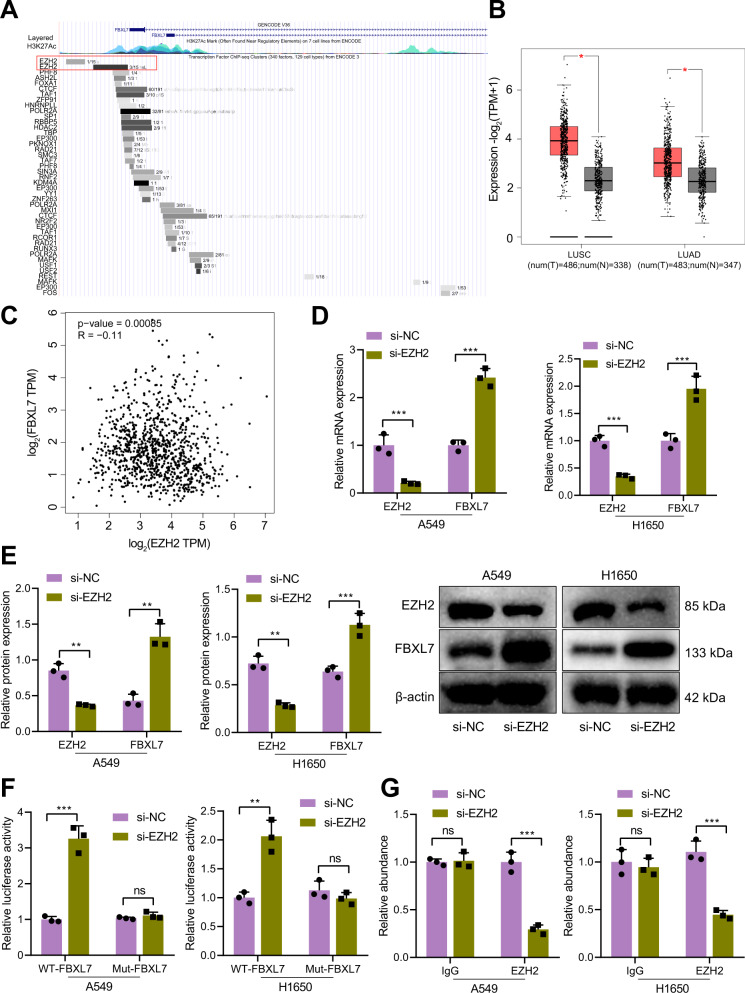
EZH2 is highly expressed in NSCLC and suppresses the transcription of FBXL7. **A** Transcriptional regulatory factors that bind to the FBXL7 promoter predicted by UCSC database, where EZH2 was found to be significantly enriched in the FBXL7 promoter region. **B** Expression of EZH2 mRNA was upregulated in NSCLC tissue samples, as analyzed by GEPIA database. Red box: tumor samples; gray box: normal samples; LUSC: lung squamous cell carcinoma; LUAD: lung adenocarcinoma. **C** Negative correlation between FBXL7 expression and EZH2 expression in NSCLC samples, as analyzed by GEPIA database. **D** FBXL7 mRNA expression was elevated in A549 and H1650 cells in the presence of si-EZH2, as determined by RT-qPCR. **E** Western blot analysis showing elevated FBXL7 protein expression in A549 and H1650 cells in the presence of si-EZH2. **F** Verification for the binding of EZH2 to the FBXL7 promoter region in A549 and H1650 cells, as determined by dual-luciferase reporter assay. **G** Enrichment of EZH2 in the promoter region of FBXL7 in A549 and H1650 cells, as observed by ChIP assay. **p < 0.01, ***p < 0.001. ns *p* > 0.05. The cell ex*p*eriment was repeated three times independently.

Next, we aimed to elucidate the upstream regulatory factors that induce the downregulation of FBXL7 in NSCLC cells. Analysis using UCSC database showed that EZH2 was significantly enriched in the FBXL7 promoter region (Fig. 5A), and GEPIA database analysis results found that EZH2 was upregulated in NSCLC samples (Fig. 5B). Correlation analysis suggested an inverse correlation between FBXL7 expression and EZH2 expression in NSCLC samples (Fig. 5C).

Meanwhile, RT-qPCR and Western blot analysis results showed upregulation of FBXL7 expression following knockdown of EZH2 in A549 and H1650 cells (Fig. 5D, E). The results of dual-luciferase reporter assay demonstrated that the luciferase activity of WT-FBXL7 was increased in si-EZH2-transduced A549 and H1650 cells while that of MUT-FBXL7 was weakened (Fig. 5F). ChIP experimental results confirmed that EZH2 was enriched in the promoter region of FBXL7, and after knockdown of EZH2, the enrichment of EZH2 in the promoter region of FBXL7 was reduced (Fig. 5G).

In summary, EZH2 diminished the expression of FBXL7 through transcription and mediated the downregulation of FBXL7 expression.

### Hypoxia induces EZH2 to downregulate FBXL7 and promote PFKFB4 protein stability

**Fig. 6:**
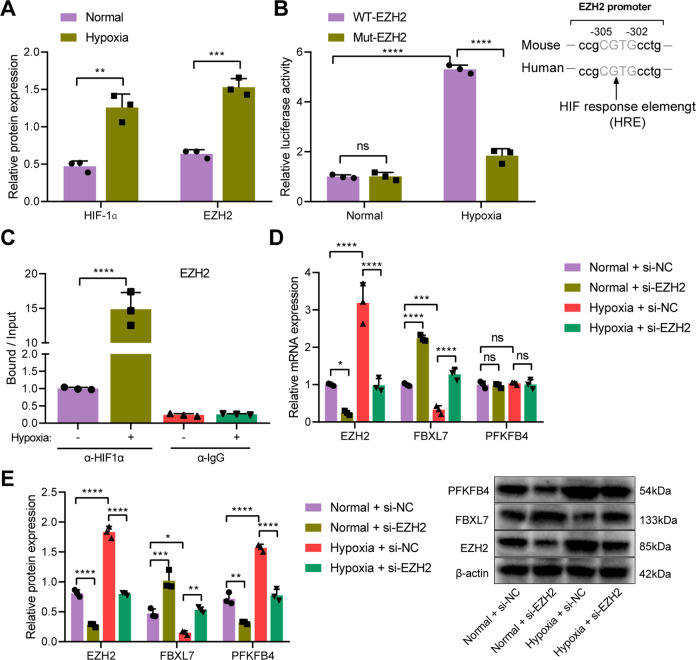
Hypoxia increases the expression of EZH2, which reduces the expression of FBXL7 and promotes PFKFB4 protein stability. **A** Western blot analysis showing elevated expression of EZH2 and HIF-1α proteins in hypoxic A549 cells. **B** Verification of the binding of HIF-1α to the EZH2 promoter region, as evaluated by dual-luciferase reporter assay. **C** Enrichment of HIF-1α in the promoter region of EZH2, as observed by ChIP assay. **D** mRNA expression of EZH2, FBXL7, and PFKFB4 in normoxic or hypoxic A549 cells in the presence of si-EZH2. **E** Western blot analysis showing the expression of EZH2, FBXL7, and PFKFB4 proteins in normoxic or hypoxic A549 cells in the presence of si-EZH2. *p < 0.05, **p < 0.01, ***p < 0.001, ****p < 0.0001. ns *p* > 0.05. The cell experiment was repeated three times independently.

Western blot analysis results showed higher expression of HIF-1α and EZH2 in A549 cells under hypoxic conditions than that under normoxic conditions (Fig. 6A). The results of dual-luciferase reporter assay revealed that luciferase activity of WT-EZH2 was enhanced in A549 cells under hypoxic conditions while that of MUT-EZH2 was weakened (Fig. 6B). ChIP assay results confirmed that under hypoxic conditions, the enrichment of HIF-1α in the promoter region of EZH2 was increased (Fig. 6C).

Subsequent RT-qPCR and Western blot analysis results presented a reduction in the expression of EZH2 yet an increase in that of FBXL7 in normoxic A549 cells transduced with si-EZH2, in addition to unchanged PFKFB4 mRNA expression and decreased protein expression. In addition, EZH2 expression was augmented, FBXL7 expression was reduced, PFKFB4 mRNA expression remained unchanged, and the protein expression was increased in hypoxia-exposed A549 cells treated with si-NC. However, si-EZH2 in hypoxia-exposed A549 cells reversed the effect of si-NC on the EZH2 and FBXL7 expression and PFKFB4 protein expression (Fig. 6D, E).

Overall, these results suggested that hypoxia induced the expression of HIF-1α, which targeted the EZH2 promoter region and promoted its transcription. Thus, EZH2 inhibited FBXL7 transcription and promoted the stability of PFKFB4 protein.

### Hypoxia promotes glucose metabolism and the malignant phenotype of NSCLC cells by regulating the EZH2/FBXL7/PFKFB4 axis

**Fig. 7:**
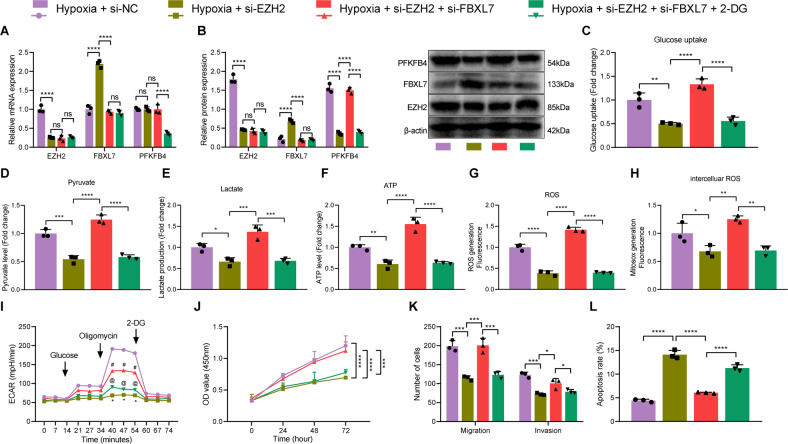
Hypoxia accelerates glucose metabolism and the malignant phenotype of NSCLC cells *via* the EZH2/FBXL7/PFKFB4 axis. **A** The mRNA expression of EZH2, FBXL7 and PFKFB4 in hypoxia-exposed A549 cells in response to si-EZH2, si-EZH2 + si-FBXL7 or si-EZH2 + si-FBXL7 + 2-DG. **B** Western blot analysis showing the expression of EZH2, FBXL7, and PFKFB4 proteins in hypoxia-exposed A549 cells in response to si-EZH2, si-EZH2 + si-FBXL7 or si-EZH2 + si-FBXL7 + 2-DG. **C** Glucose uptake measurement in response to si-EZH2, si-EZH2 + si-FBXL7, or si-EZH2 + si-FBXL7 + 2-DG. **D** Pyruvate level measurement in response to si-EZH2, si-EZH2 + si-FBXL7, or si-EZH2 + si-FBXL7 + 2-DG. **E** Lactate production measurement in response to si-EZH2, si-EZH2 + si-FBXL7, or si-EZH2 + si-FBXL7 + 2-DG. **F** ATP level measurement in response to si-EZH2, si-EZH2 + si-FBXL7, or si-EZH2 + si-FBXL7 + 2-DG. **G** ROS content measurement in A549 cells in response to si-EZH2, si-EZH2 + si-FBXL7 or si-EZH2 + si-FBXL7 + 2-DG. **H** ROS content measurement in mitochondrion in response to si-EZH2, si-EZH2 + si-FBXL7, or si-EZH2 + si-FBXL7 + 2-DG. **I** Glycolysis of A549 cells in response to si-EZH2, si-EZH2 + si-FBXL7, or si-EZH2 + si-FBXL7 + 2-DG, as detected by ECAR assay (^#^*p* < 0.05 vs. the Hypoxia + si-EZH2 group; ^@^*p* < 0.05 vs. the Hypoxia + si-NC + si-FBXL7 group). **J** Viability of A549 cells in response to si-EZH2, si-EZH2 + si-FBXL7 or si-EZH2 + si-FBXL7 + 2-DG, as analyzed by CCK-8 assay. **K** Migration and invasion of A549 cells, as analyzed by Transwell assay. **L** Apoptosis of A549 cells in response to si-EZH2, si-EZH2 + si-FBXL7, or si-EZH2 + si-FBXL7 + 2-DG, as analyzed by flow cytometry. *p < 0.05, **p < 0.01, ***p < 0.001, ****p < 0.0001. ns *p* > 0.05. The cell experiment was repeated three times independently.

We then proceeded to explore the effect of hypoxia-mediated EZH2/FBXL7/PFKFB4 axis on the glycolysis. RT-qPCR and Western blot analysis detection results showed that under hypoxic conditions, cells transduced with si-EZH2 exhibited decreased expression of EZH2, increased expression of FBXL7, unchanged mRNA expression of PFKFB4, and reduced PFKFB4 protein expression. Further si-FBXL7 abolished the effect of si-EZH2 on EZH2 and FBXL7 expression and PFKFB4 protein expression. Treatment with 2-DG did not alter EZH2 and FBXL7 expression while decreasing PFKFB4 mRNA and protein expression (Fig. 7A, B).

In addition, transduction with si-EZH2 reduced glucose uptake, pyruvate level, ATP level, lactate production, and ROS content while si-EZH2 + si-FBXL7 resulted in contrasting results. Further treatment with 2-DG brought about the same results as those of si-EZH2 (Fig. 7C–H).

ECAR results in Fig. 7I demonstrated that the glycolysis was weakened upon silencing of EZH2, which was restored upon silencing of FBXL7. In the presence of si-EZH2 + si-FBXL7 + 2-DG, glycolysis was attenuated. In addition, silencing of EZH2 was found to impair cell viability, migration, and invasion while inducing cell apoptosis; however, opposite results were noted in the absence of FBXL7. Further treatment with 2-DG caused the consistent results with those of si-EZH2 (Fig. 7J–L).

Altogether, knockdown of EZH2 can promote the transcription of FBXL7, which in turn reduced the expression of PFKFB4 protein, thereby inhibiting the glucose metabolism of NSCLC cells, and repressing cell malignant phenotype.

### EZH2 facilitates tumor growth in vivo by regulating FBXL7/PFKFB4

**Fig. 8:**
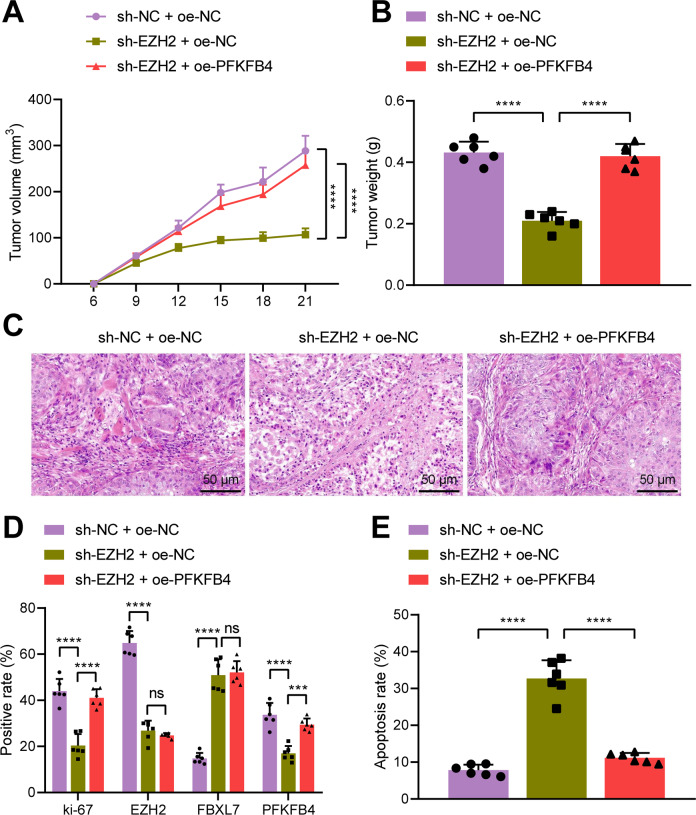
Knockdown of EZH2 retards tumor growth in vivo *via* the FBXL7/PFKFB4 axis. **A** Quantitative analysis for tumor volume of nude mice subcutaneously inoculated with cells transduced with sh-EZH2 alone or combined with oe-PFKFB4. **B** Quantitative analysis for tumor weight of nude mice subcutaneously inoculated with cells transduced with sh-EZH2 alone or combined with oe-PFKFB4. **C** HE staining analysis of the tumor tissues of nude mice subcutaneously inoculated with cells transduced with sh-EZH2 alone or combined with oe-PFKFB4. **D** Immunohistochemical staining analysis of Ki-67, EZH2, PFKFB4, and FBXL7 proteins in the tumor tissues of nude mice subcutaneously inoculated with cells transduced with sh-EZH2 alone or combined with oe-PFKFB4. **E** TUNEL-positive cells in the tumor tissues of nude mice subcutaneously inoculated with cells transduced with sh-EZH2 alone or combined with oe-PFKFB4. ***p < 0.001, ****p < 0.0001. ns *p* > 0.05. *n* = 6 for mice in each group.

Finally, we aimed to characterize the effect of EZH2/FBXL7/PFKFB4 axis on NSCLC in nude mice. Tumor volume and weight of mice treated with sh-EZH2 + oe-NC were observed to be reduced while they were increased in the presence of sh-EZH2 + oe-PFKFB4 (Fig. 8A, B).

Analysis on the tumor tissues of mice using HE staining found obvious inflammatory cell infiltration in mice treated with sh-NC + oe-NC but silencing of EZH2 reduced inflammatory cell infiltration. sh-EZH2 + oe-PFKFB4 restored the increase of inflammatory cell infiltration (Fig. 8C). In addition, immunohistochemical staining results suggested a decline in the Ki-67, EZH2, and PFKFB4 positive cells yet an increase in FBXL7 positive cells upon silencing of EZH2. Further overexpression of PFKFB4 increased Ki-67 and PFKFB4 positive cells without altering EZH2 and FBXL7 positive cells (Fig. 8D). TUNEL data exhibited that apoptosis rate was augmented following EZH2 silencing, the effect of which was reversed following PFKFB4 overexpression (Fig. 8E).

Taken together, knockdown of EZH2 inhibited tumor growth in vivo by mediating the FBXL7/PFKFB4 axis.

## Discussion

Hypoxia is frequently present in lung cancer and associated with the cancer progression, metastasis, and metabolism [33]. The findings collected from this study supported the promoting effect of hypoxia on the glucose metabolism and tumor growth of NSCLC *via* regulation of the EZH2/FBXL7/PFKFB4 axis.

The current study first revealed that FBXL7 was poorly expressed in NSCLC tissue samples and cells, and its downregulation was predictive of poor overall survival of patients with NSCLC. In contrast, FBXL7 overexpression could inhibit the malignant phenotypes of NSCLC cells. Partially in line with this, low FBXL7 mRNA expression indicates poor survival in patients with pancreatic and prostatic cancers; this low expression can also facilitate the invasion of pancreatic and prostatic cancers [34]. Here, the present study revealed that FBXL7 promoted the ubiquitination-mediated degradation of PFKFB4 which was highly expressed in NSCLC tissue samples. Increasing studies have demonstrated the promoting effect of PFKFB4 on the ubiquitination-mediated degradation of several factors, including survivin, c-SRC, and Snail1, thus decreasing their protein expression [16, 18, 34]. PFKFB4 expression is increased in lung adenocarcinoma and this increase can promote the oncogenic phenotypes of lung adenocarcinoma cells [35]. PFKFB4 plays important roles in tumor cell glucose metabolism as it shows more FBPase-2 activity, redirecting glucose to the pentose phosphate pathway, and supporting ROS detoxification and lipid and nucleotide synthesis [36]. FBXL7 is a newly identified SCF E3 ubiquitin ligase that induces ubiquitination of target proteins, leading to proteasome-dependent degradation [16, 37]. Our results showed that FBXL7 knockdown did not affect PFKFB4 mRNA levels. Therefore, it was speculated that FBXL7 may interact with PFKFB4, leading to the ubiquitination and degradation of PFKFB4. The results of Co-IP and GST-pull down assays confirmed that PFKFB4 was a substrate of the ubiquitin ligase FBXL7, and FBXL7 degraded PFKFB4 protein through ubiquitination. In addition, the degradation rate of PFKFB4 protein was accelerated in the FBXL7 presence, indicating that FBXL7 enhanced the ubiquitination of PFKFB4 protein. Collectively, these data can support the conclusion that FBXL7 reduced the expression of PFKFB4, thereby arresting glucose metabolism and the resultant malignant phenotype of NSCLC cells.

Further analysis exhibited that EZH2 was highly expressed in NSCLC tissue samples and inhibited the transcription of FBXL7. EZH2 has been involved in transcriptional repression, mainly targeting tumor-suppressor genes and thus promoting tumorigenesis [38, 39]. Silencing of EZH2 can also significantly inhibit the oncogenic phenotypes of A549 cells [40]. In addition, EZH2 silencing in prostate cancer cell experiments inhibits glucose metabolism process [41]. Furthermore, the current results demonstrated that hypoxia enhanced glucose metabolism and the malignant phenotype of NSCLC cells by regulating the EZH2/FBXL7/PFKFB4 axis. Specifically, hypoxia induced the expression of HIF-1α, which targeted the EZH2 promoter region and promoted its transcription. EZH2 inhibited FBXL7 transcription and promoted the stability of PFKFB4 protein, thus enhancing glucose metabolism and the malignant phenotype of NSCLC cells. Hypoxia powers the mechanisms underlying cancer progression, especially lung cancer; hypoxia-induced effects are orchestrated by hypoxia-inducible factors (HIFs) that regulate expression of multiple genes involved in cancer progression [42]. In accordance with the present results, hypoxia exposure can increase the expression of HIF-1α which can bind to the EZH2 promoter and result in increased EZH2 expression [43–45].

**Fig. 9:**
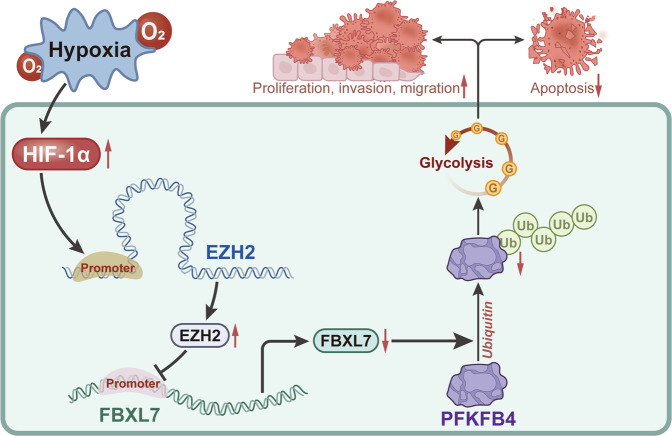
Schematic mechanism of hypoxia-mediated EZH2/FBXL7/PFKFB4 axis in NSCLC. Hypoxia induces the expression of HIF-1α in NSCLC cells. HIF-1α elevates expression of EZH2 which in turn reduces expression of FBXL7, and inhibits the ubiquitination of PFKFB4 by FBXL7, resulting in upregulation of PFKFB4 protein expression. By this mechanism, NSCLC cell glycolysis and malignant phenotypes are accelerated while cell apoptosis was decelerated.

Overall, our study indicates that hypoxia can promote the glycolysis and tumor growth of NSCLC by regulating the EZH2/FBXL7/PFKFB4 axis (Fig. 9). This novel axis can provide better understanding of the growth and metastasis of NSCLC. However, mutant p53 interacts with EZH2 and enhances its association with the chromatin for epigenetic regulation. A previous study has shown that the dihydrofolate reductase (DHFR) inhibitor methotrexate (MTX) initiates p53-dependent apoptosis and restores E-cadherin expression by downregulating HDAC/EZH2, which subsequently curtails NSCLC progression [46]. Therefore, we speculated that, in addition to the existing regulatory mechanisms involved in this study, hypoxia may also inhibit p53-dependent apoptosis and promote NSCLC progression by inducing HIF-1α expression and up-regulating EZH2 expression in NSCLC cells. Future studies based on in vitro cell experiments of the p53 mutant cell line are warranted.

## Supplementary information

Supplementary Tables

aj-checklist

WB Figures

## Data Availability

All data generated or analyzed during this study are included in this article and its supplementary material files. Further enquiries can be directed to the corresponding authors.
