# Supplementary material for: Hypoxia-mediated promotion of glucose metabolism in non-small cell lung cancer correlates with activation of the EZH2/FBXL7/PFKFB4 axis
Source: Cell Death Dis. 2023 May 13;14(5):326. doi: 10.1038/s41419-023-05795-z (PMC10182982; doi:10.1038/s41419-023-05795-z)
Supplement: Supplementary file 1 — Supplementary Tables [file 41419_2023_5795_MOESM1_ESM.docx]

**Supplementary Table 1** Primer sequences for reverse transcription quantitative polymerase chain reaction

| Gene | Primer sequence |
| --- | --- |
| EZH2 | Forward 5’-AATCAGAGTACATGCGACTGAGA-3’ |
|  | Reverse 5’-GCTGTATCCTTCGCTGTTTCC-3’ |
| FBXL7 | Forward 5’-GATCACACGCCCACTAAAGC-3’ |
|  | Reverse 5’-CCTTCCATTCTGAAATCCTGGG-3’ |
| PFKFB4 | Forward 5’-GGGTGCCTCTTGGCCTTAAA-3’ |
|  | Reverse 5’-GCCCACACGGCATACTTTTC-3’ |
| Actin | Forward 5’-CTCACCATGGATGATGATATCGC-3’ |
|  | Reverse 5’-AGGAATCCTTCTGACCCATGC-3’ |

**Supplementary Table 2** Identification of FBXL7 mutual substrate protein by TAP-MS

| Protein | Molecular weight | Number of identified peptides | Proportion of full-length sequence covered by peptides (%) |
| --- | --- | --- | --- |
| FBXL7 | 54268 | 18 | 37.8 |
| RUVBL2 | 48246 | 13 | 18.2 |
| STIP1 | 67521 | 12 | 14.1 |
| ATP5A1 | 56320 | 9 | 12.5 |
| WDR48 | 72649 | 9 | 11.4 |
| GMPPA | 44852 | 5 | 11.9 |
| PFKFB4 | 52690 | 7 | 11.5 |
| HMGCS1 | 60243 | 5 | 10.2 |
| MLX | 35218 | 4 | 9.8 |
| AURKA | 42596 | 5 | 9.2 |
| CLCF1 | 26445 | 3 | 9.9 |
| FGD2 | 71492 | 4 | 5.8 |
| GRWD1 | 51103 | 3 | 6.2 |
