# Supplementary material for: Hypoxia-mediated promotion of glucose metabolism in non-small cell lung cancer correlates with activation of the EZH2/FBXL7/PFKFB4 axis
Source: Cell Death Dis. 2023 May 13;14(5):326. doi: 10.1038/s41419-023-05795-z (PMC10182982; doi:10.1038/s41419-023-05795-z)

Fig. 1K-1


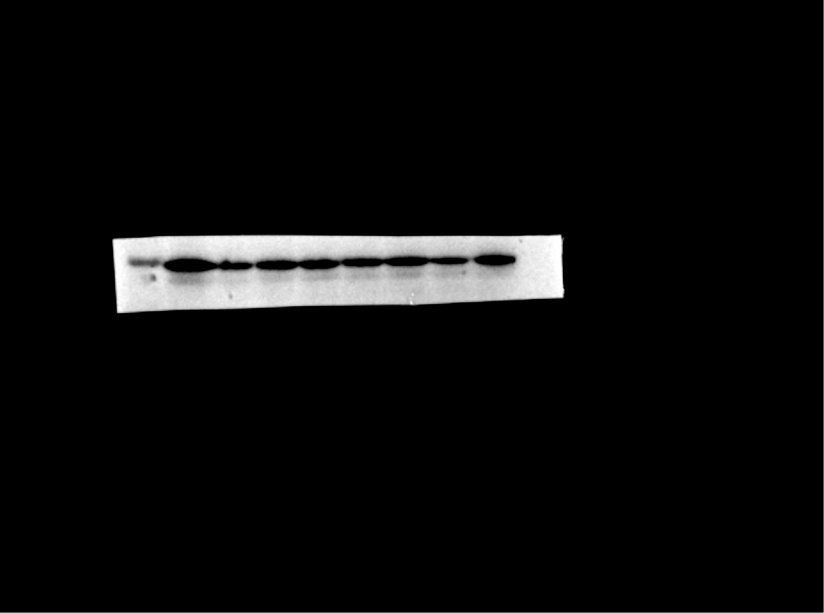


Fig. 1K-2


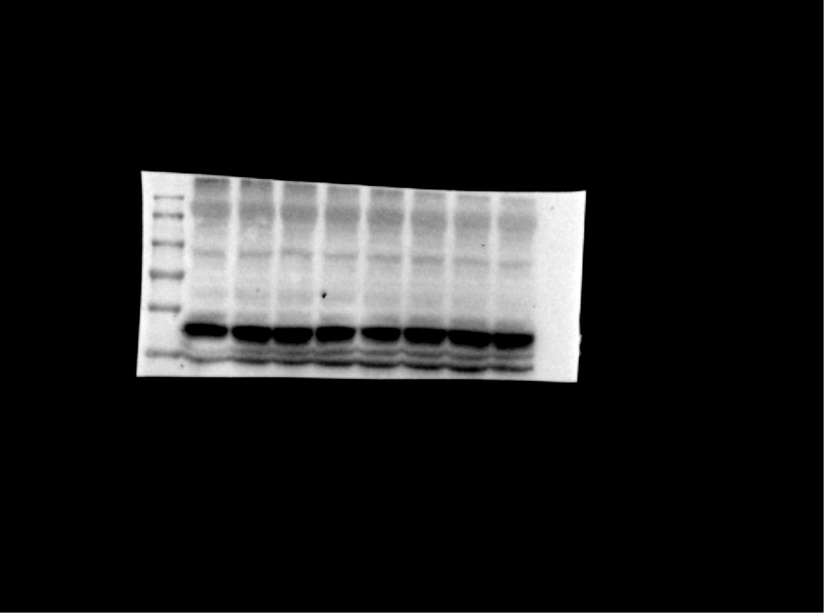


Fig. 2B-1


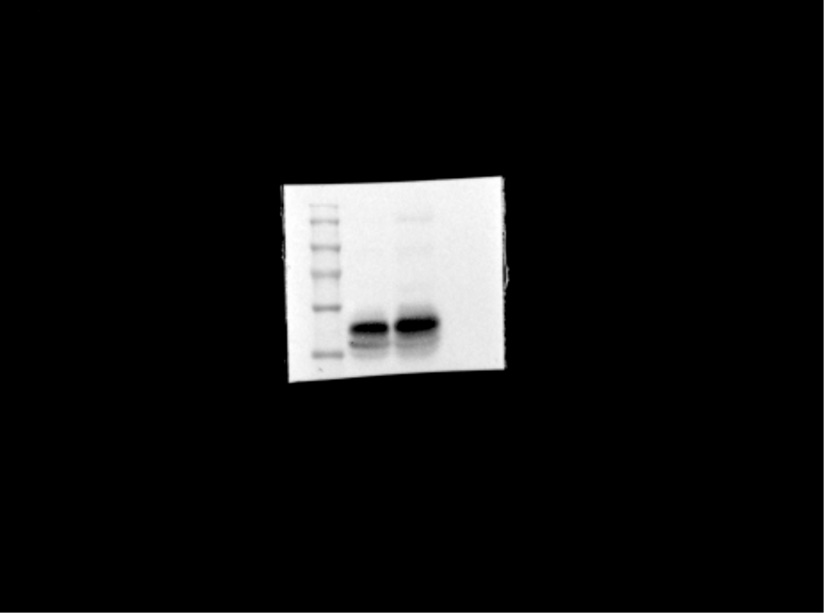


Fig. 2B-2


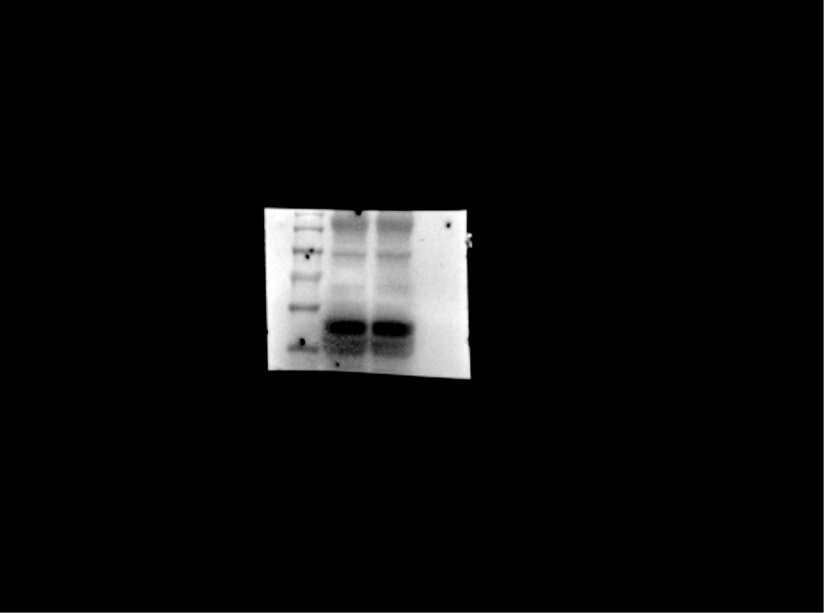


Fig. 2B-3


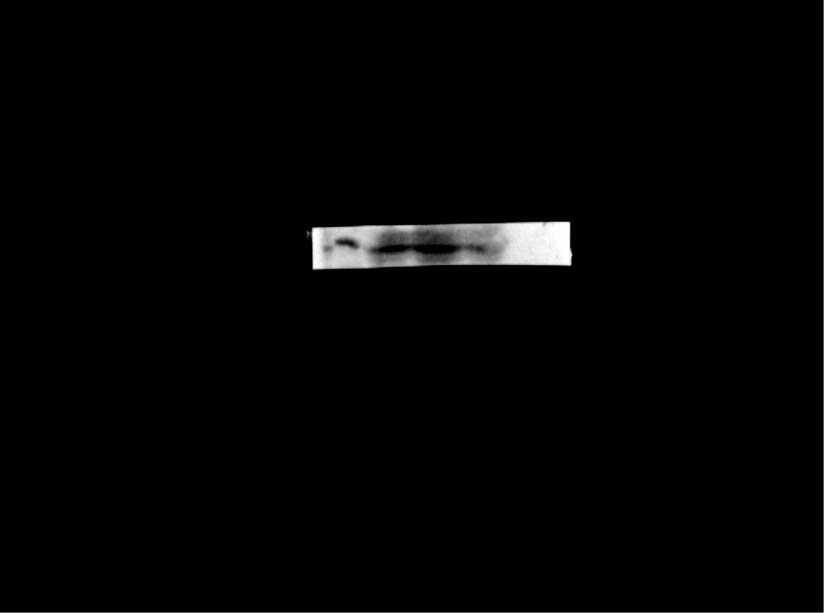


Fig. 2B-4


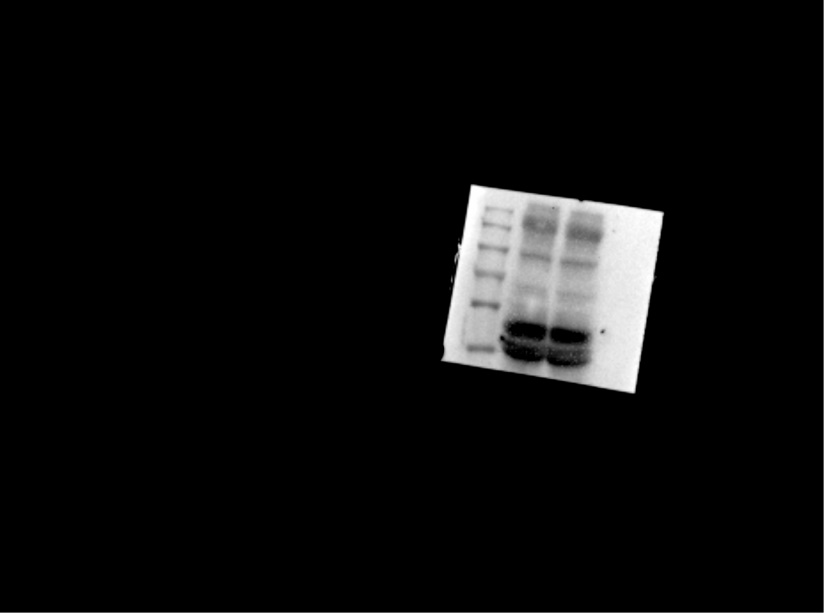


Fig. 3C-1


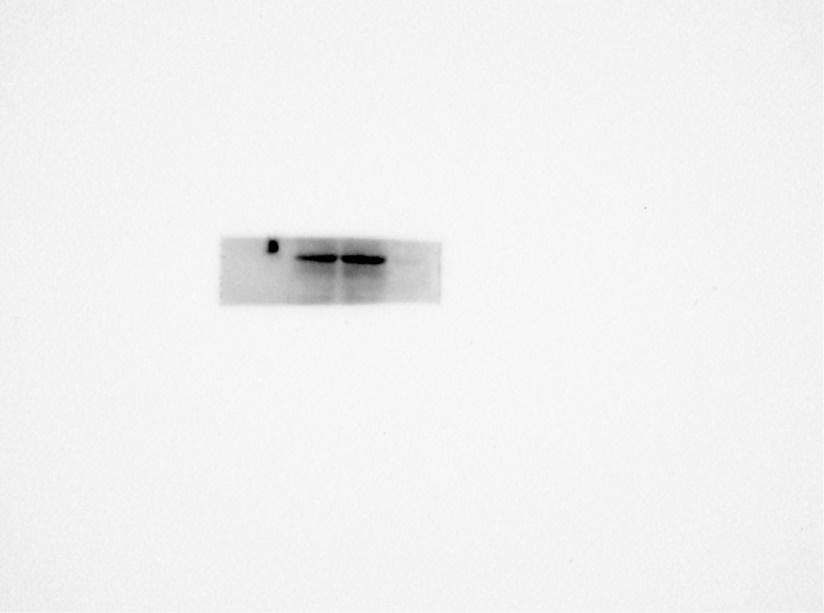


Fig. 3C-2


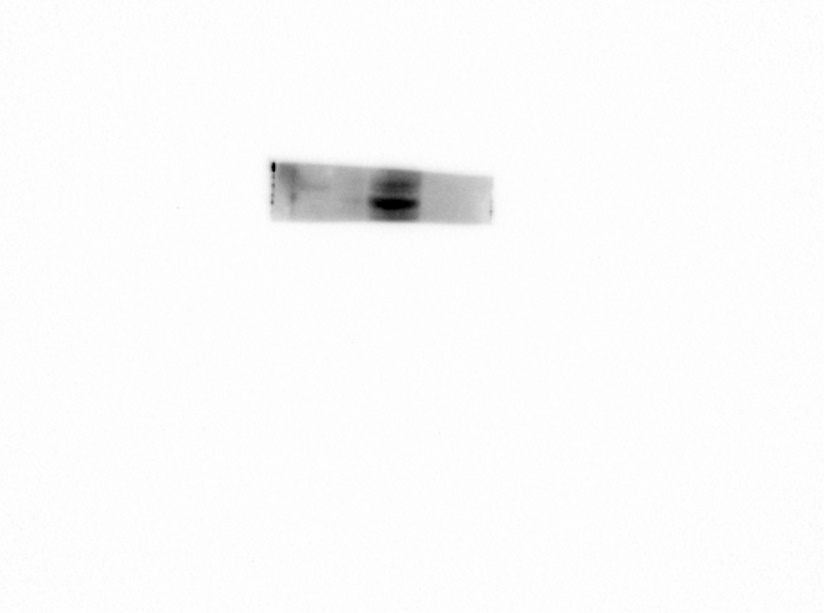


Fig. 3C-3


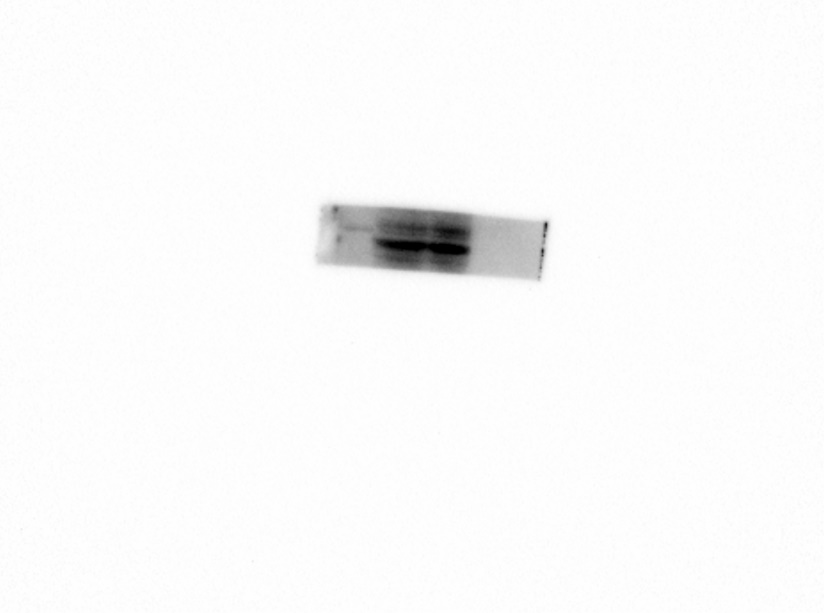


Fig. 3C-4


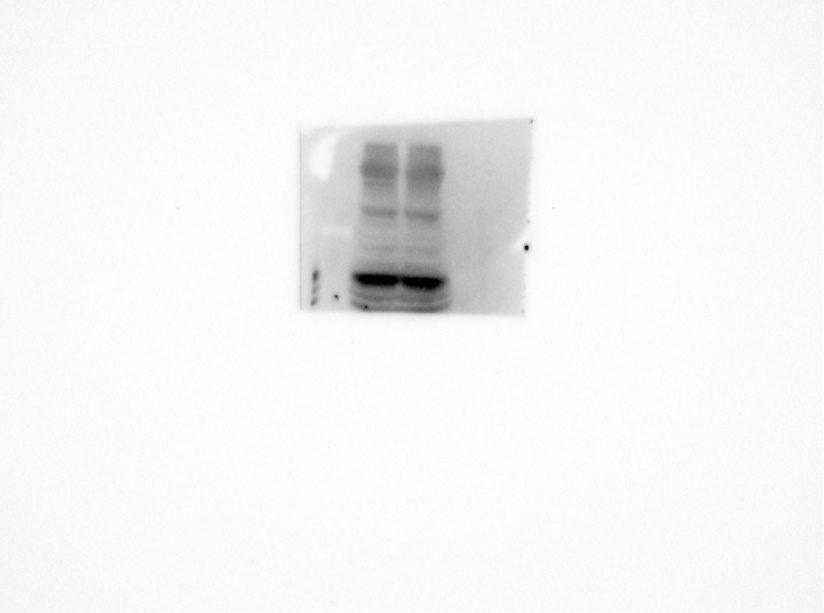


Fig. 3D-1


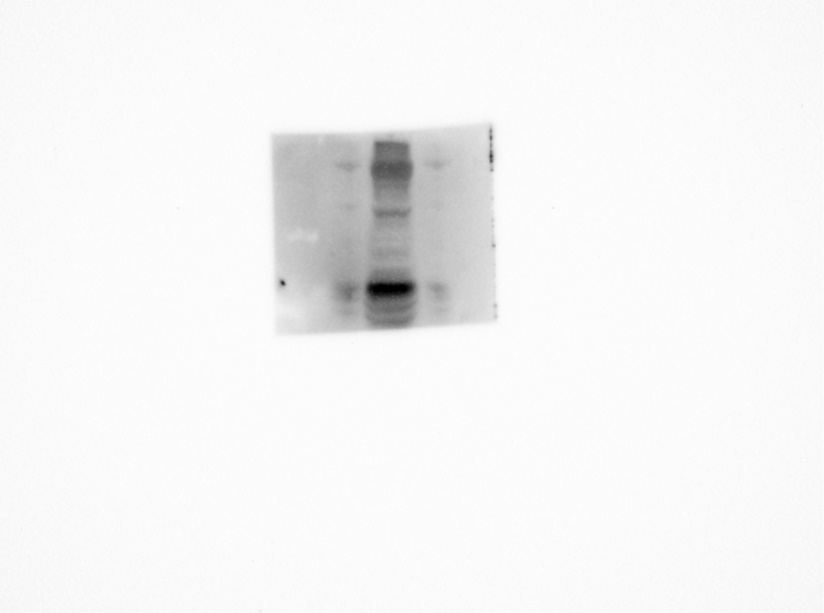


Fig. 3D-2


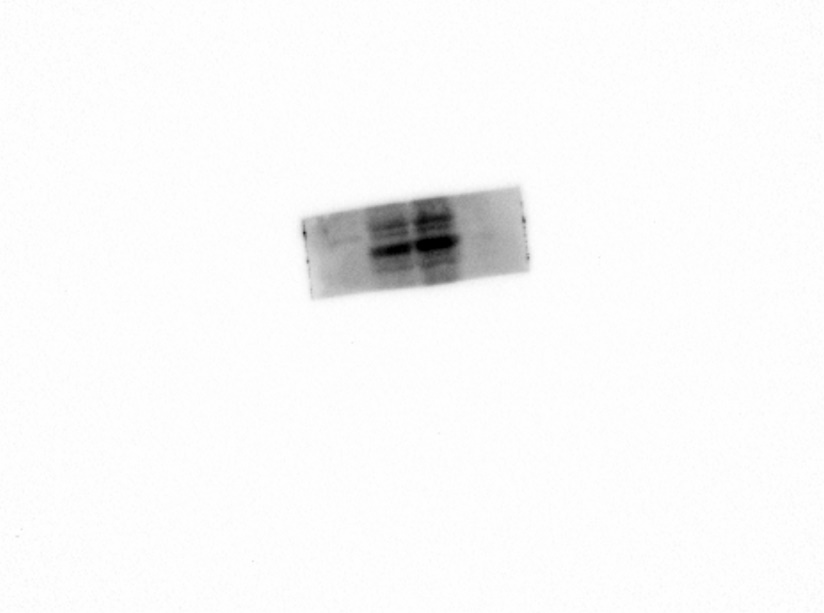


Fig. 3D-3


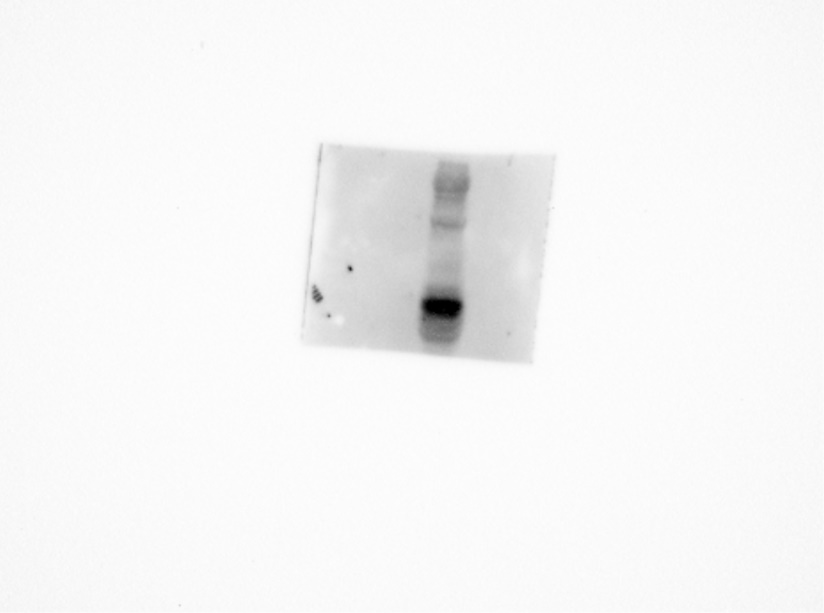


Fig. 3D-4


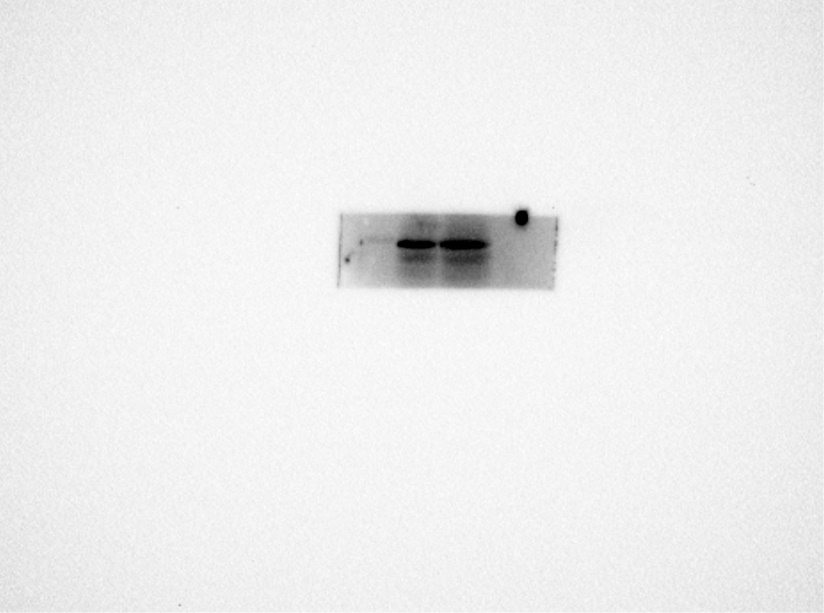


Fig. 3D-5


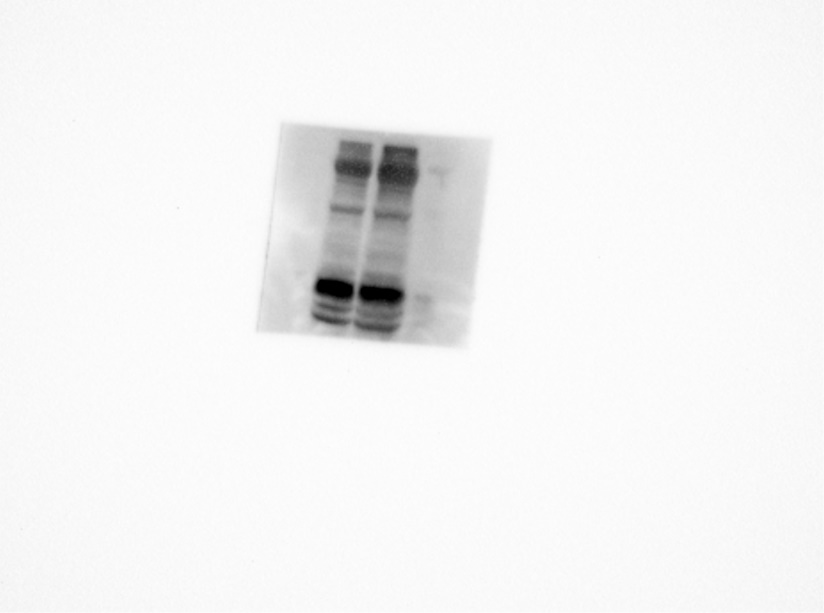


Fig. 3D-6


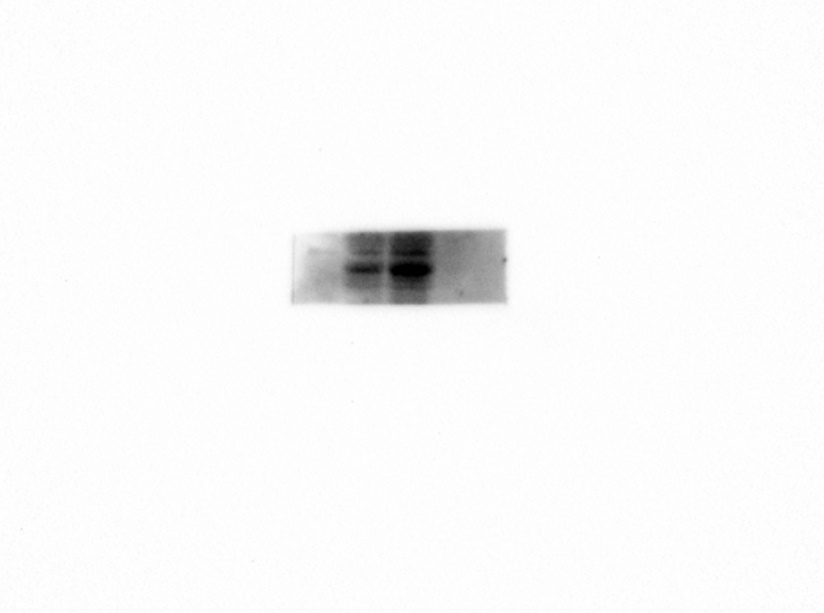


Fig. 3D-7


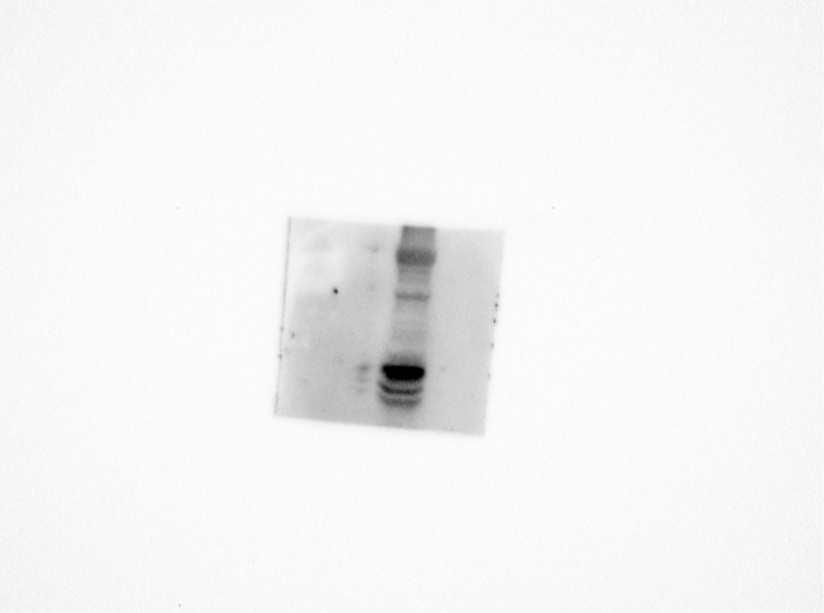


Fig. 3D-8


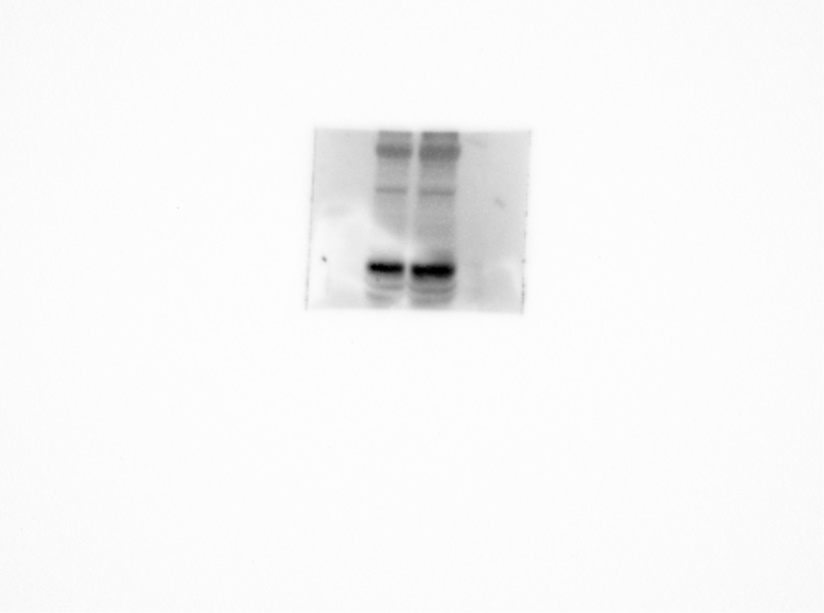


Fig. 3D-9


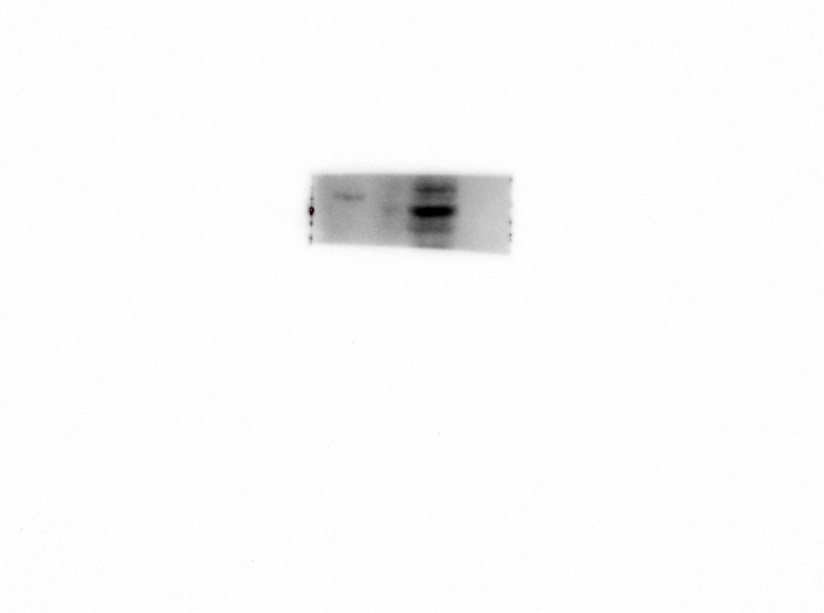


Fig. 3D-10


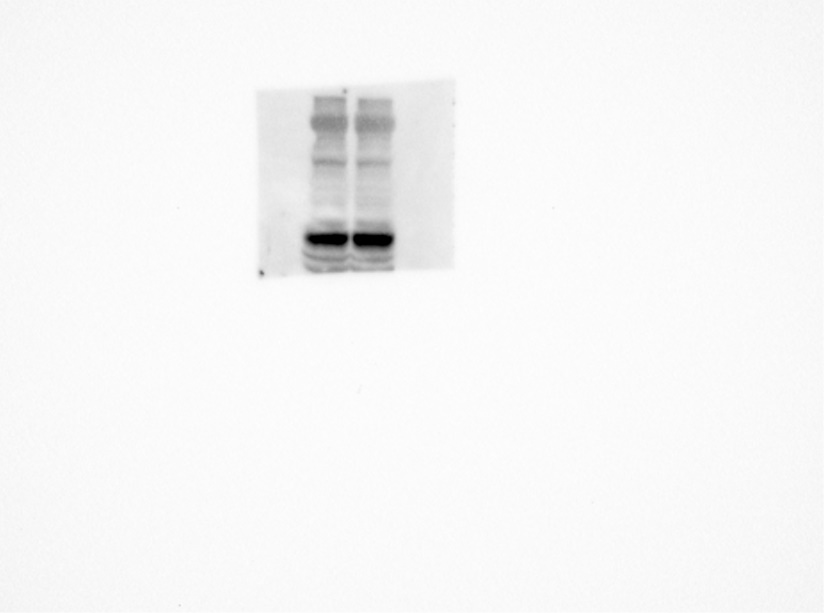


Fig. 3E-1


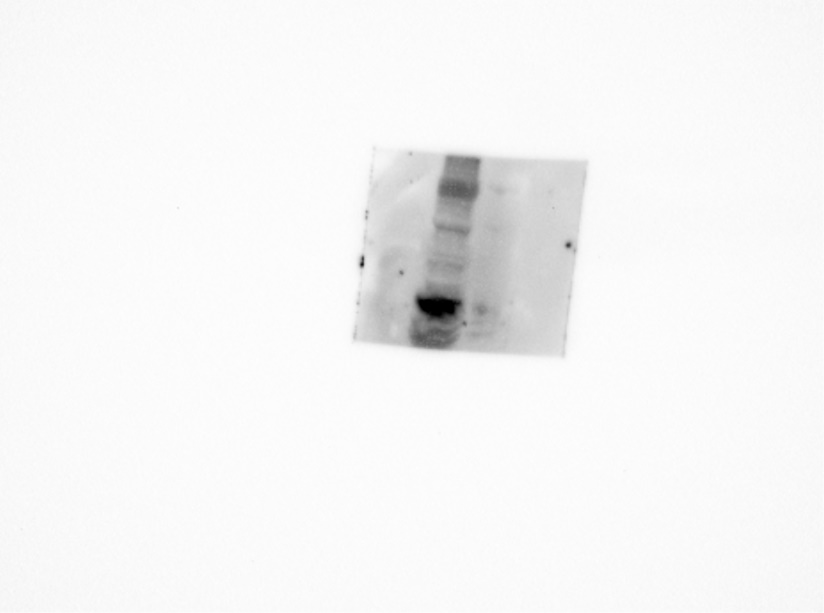


Fig. 3E-2


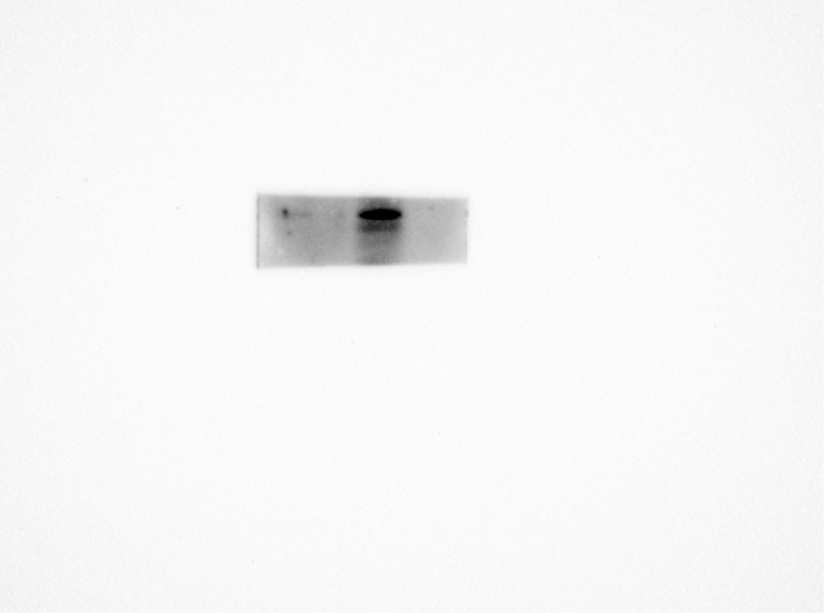


Fig. 3E-3


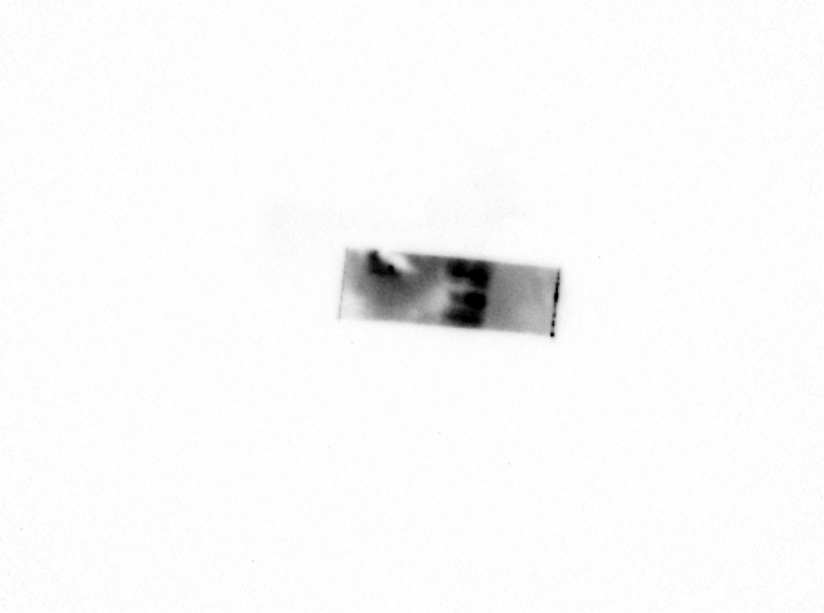


Fig. 3G-1


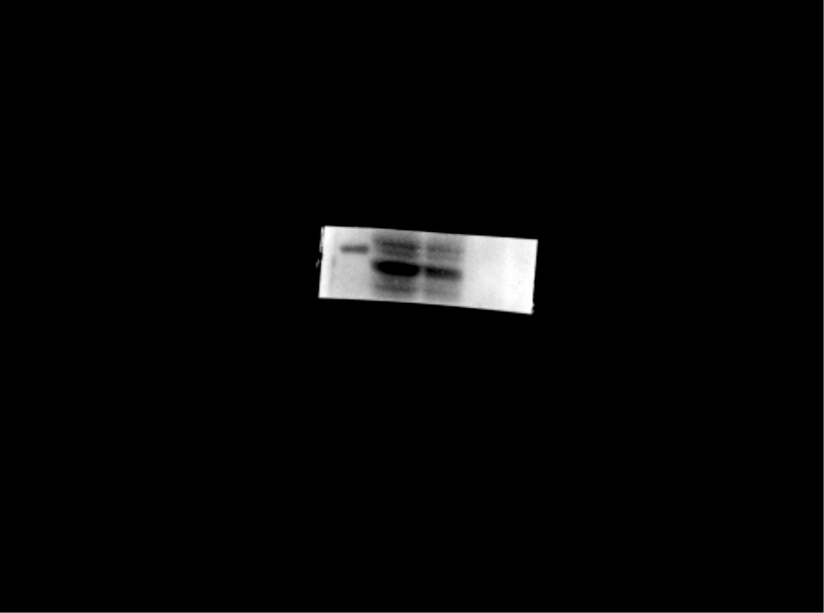


Fig. 3G-2


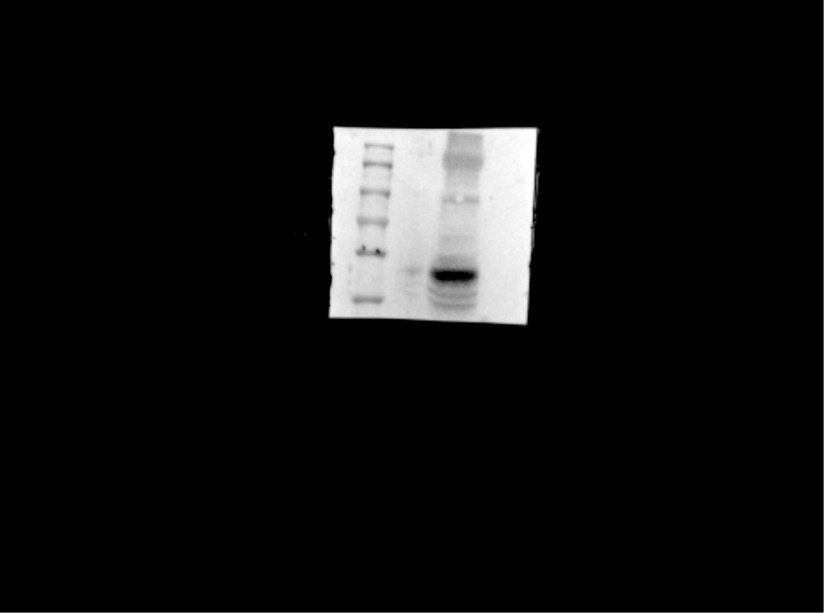


Fig. 3G-3


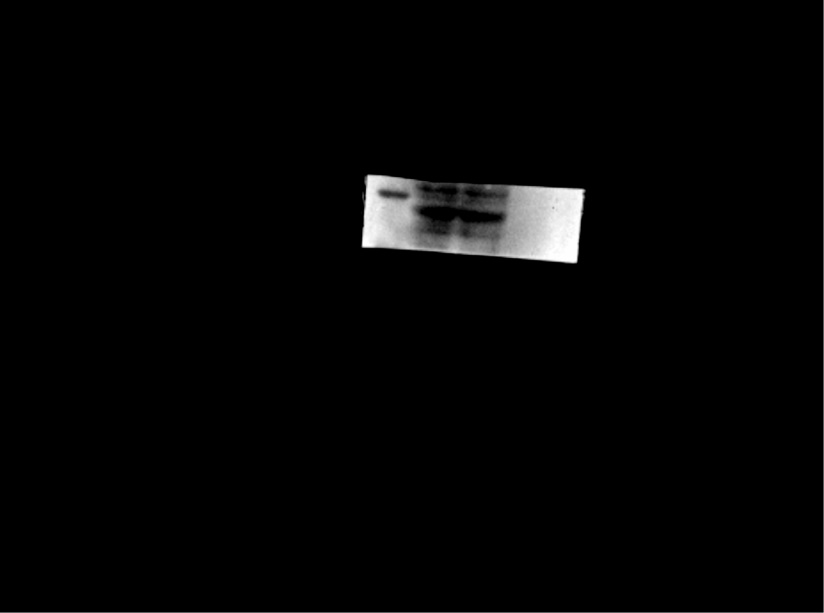


Fig. 3I-1


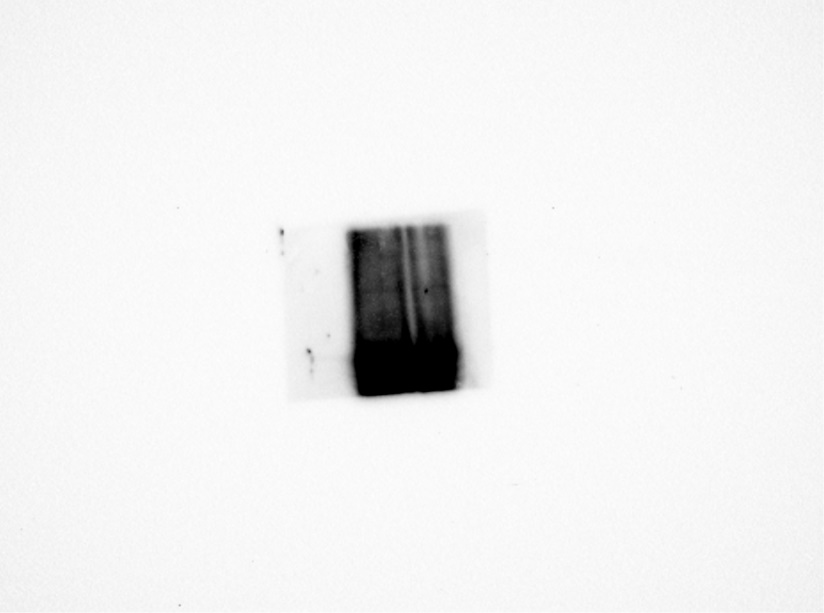


Fig. 3I-2


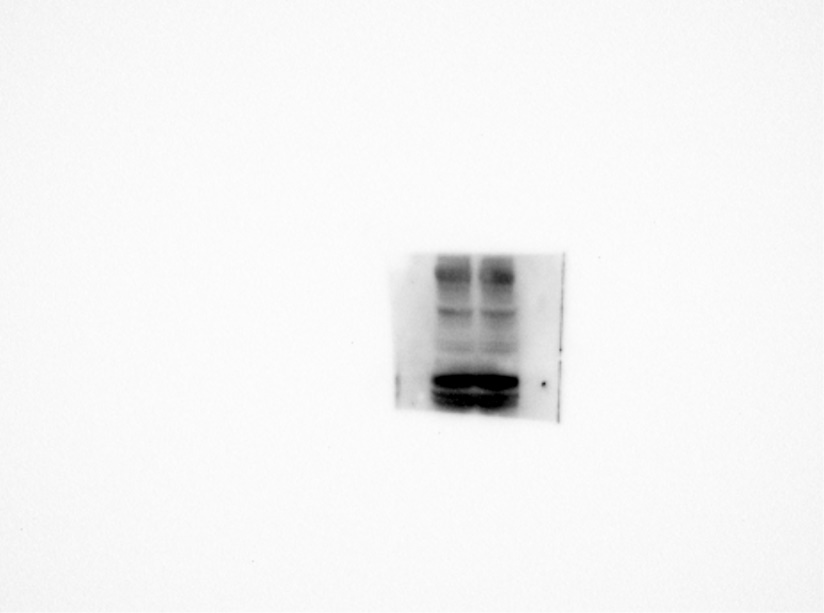


Fig. 3I-3


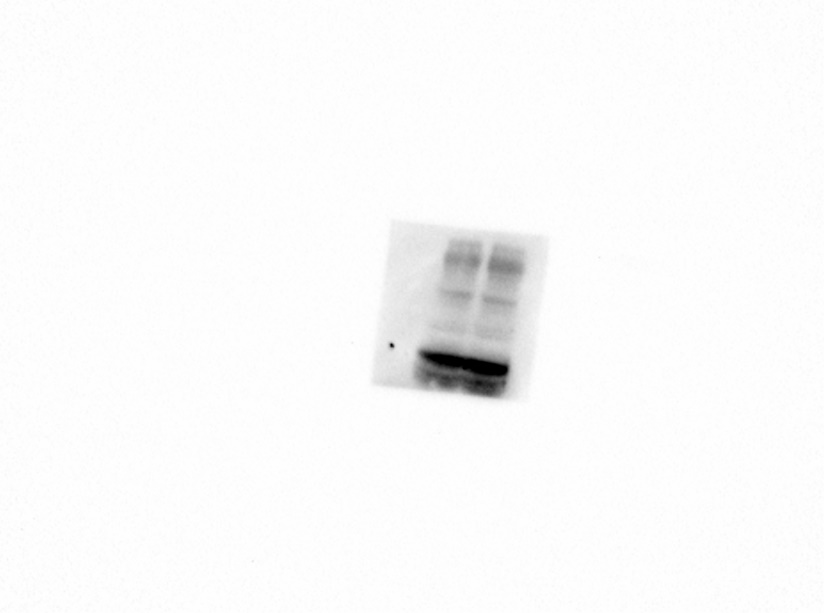


Fig. 3I-4


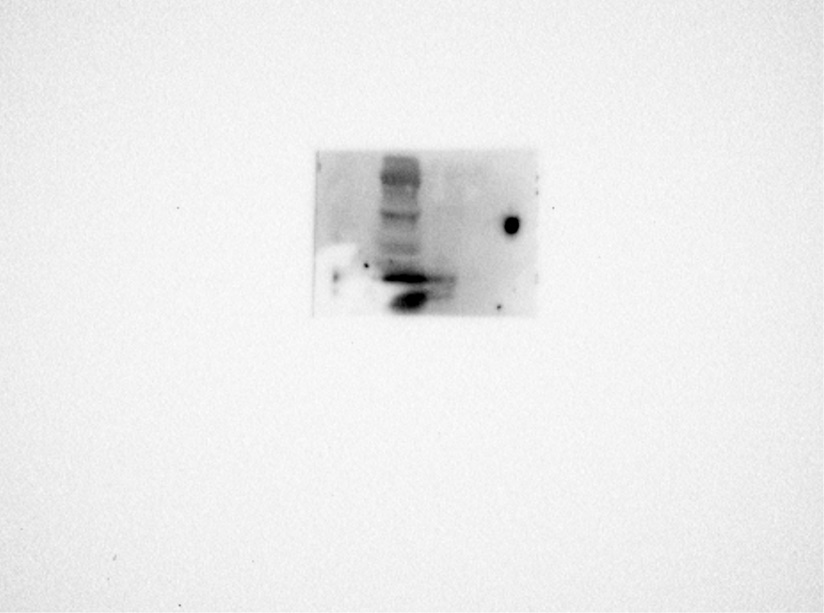


Fig. 3I-5


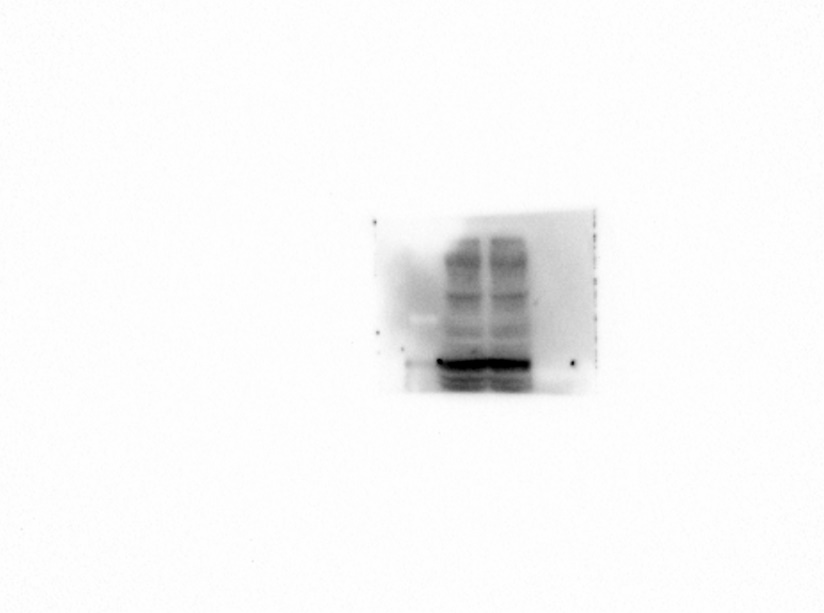


Fig. 3J-1


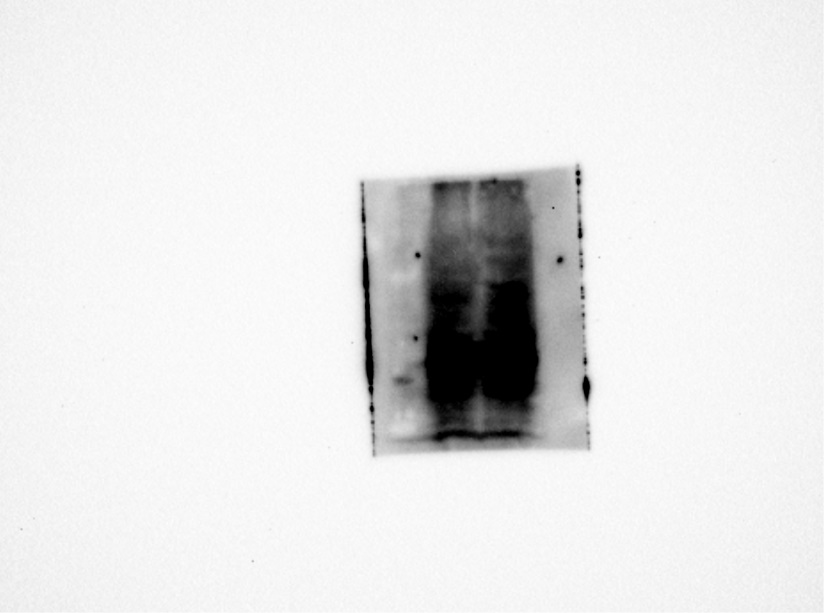


Fig. 3J-2


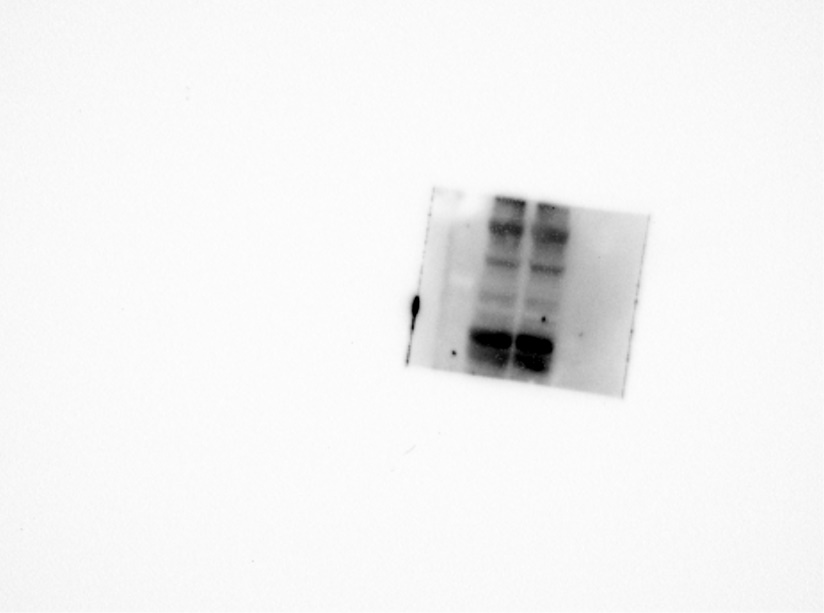


Fig. 3J-3


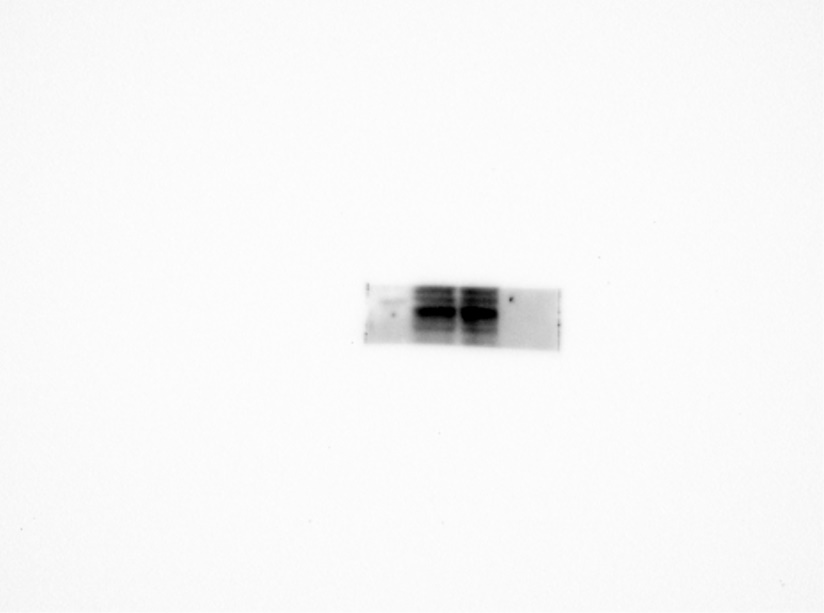


Fig. 3J-4


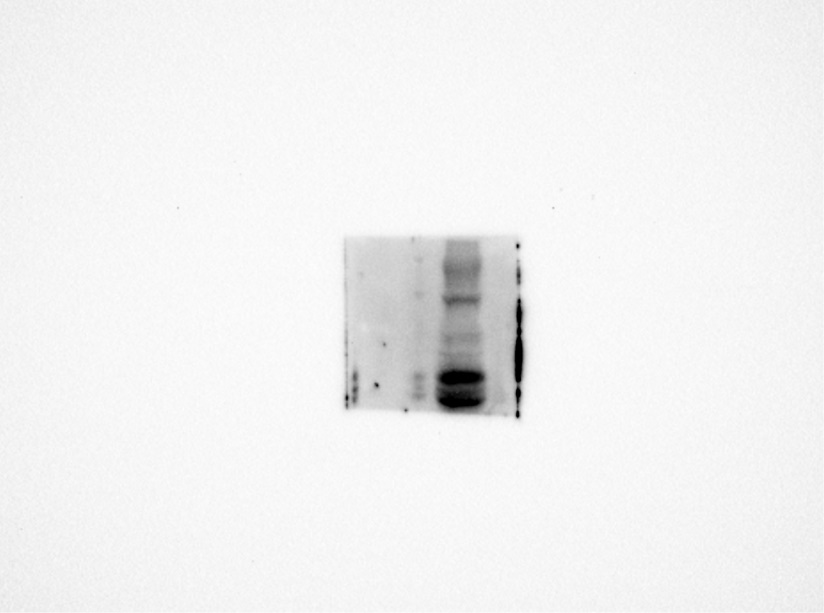


Fig. 3J-5


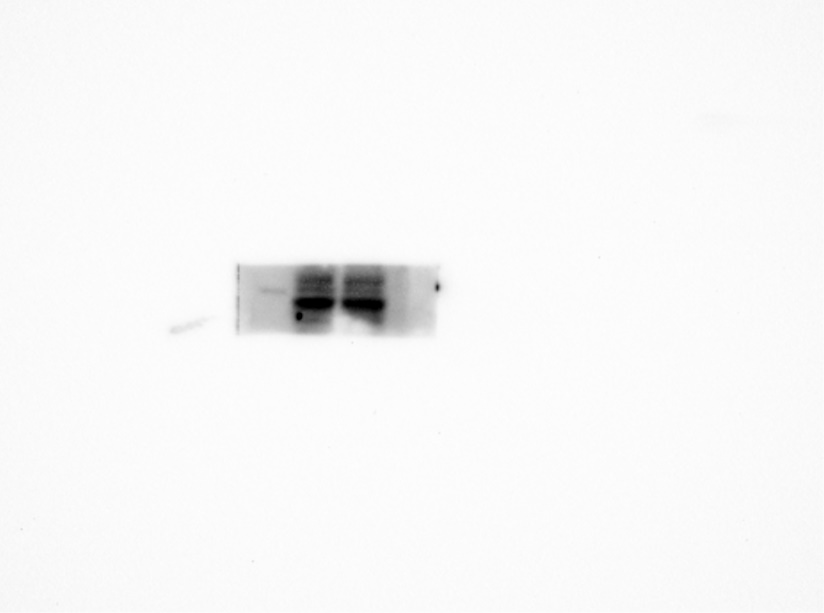


Fig. 4A-1


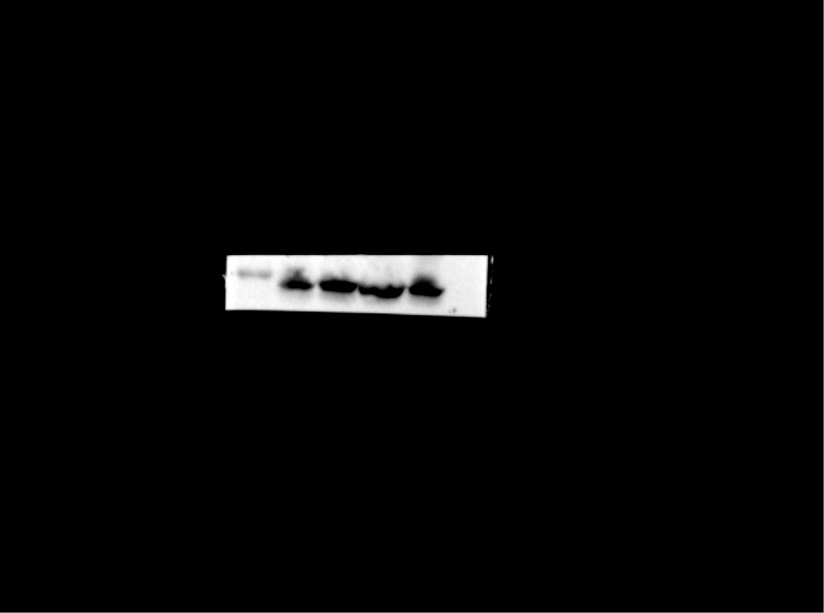


Fig. 4A-2


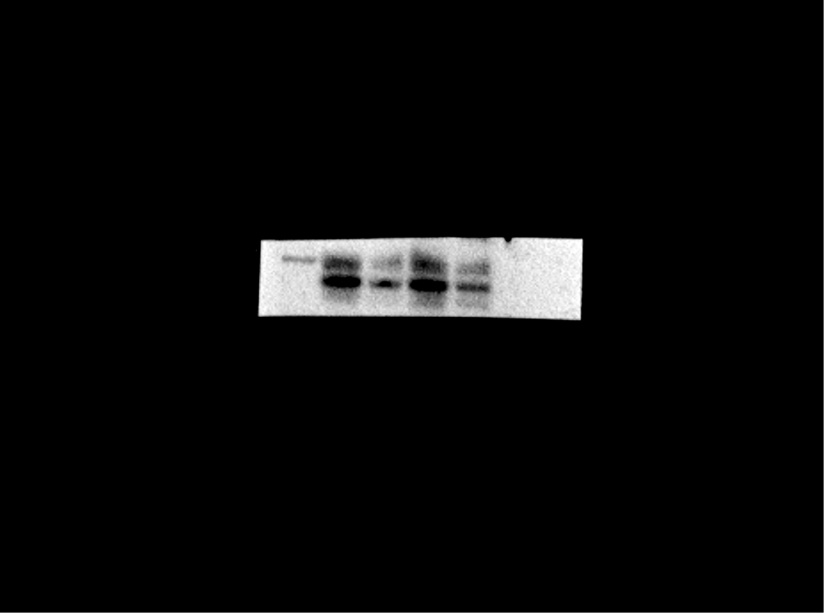


Fig. 4A-3


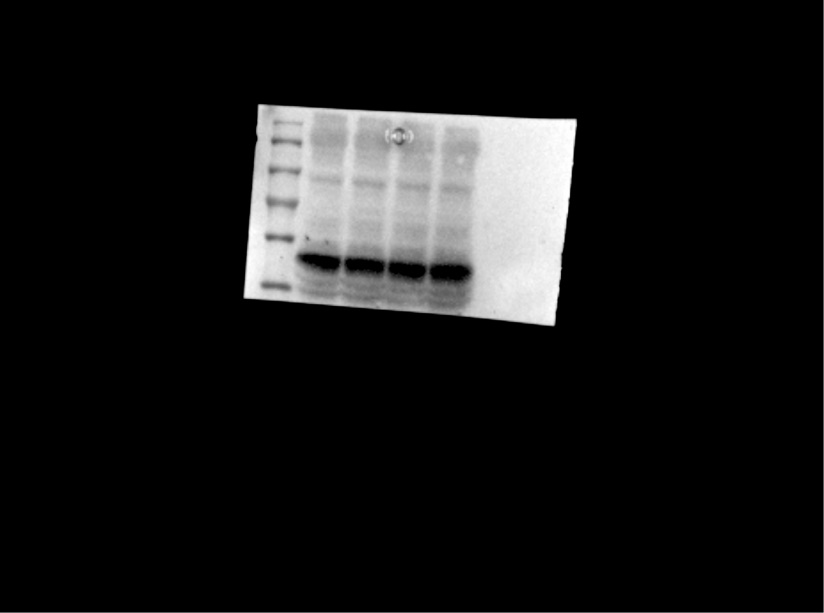


Fig. 5E-1


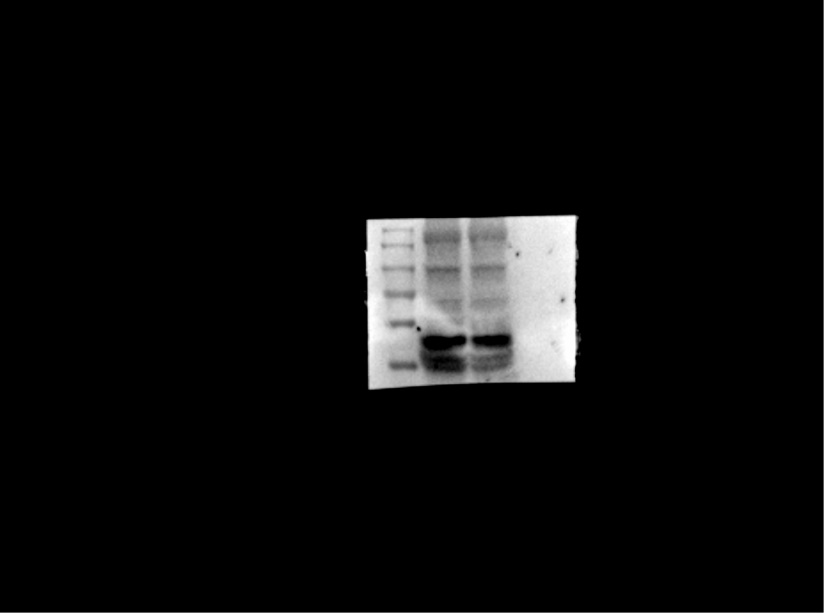


Fig. 5E-2


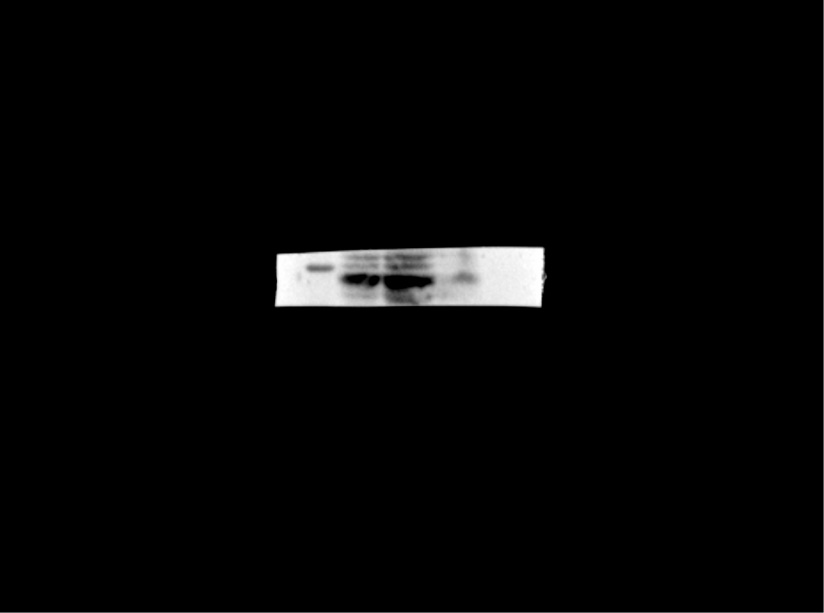


Fig. 5E-3


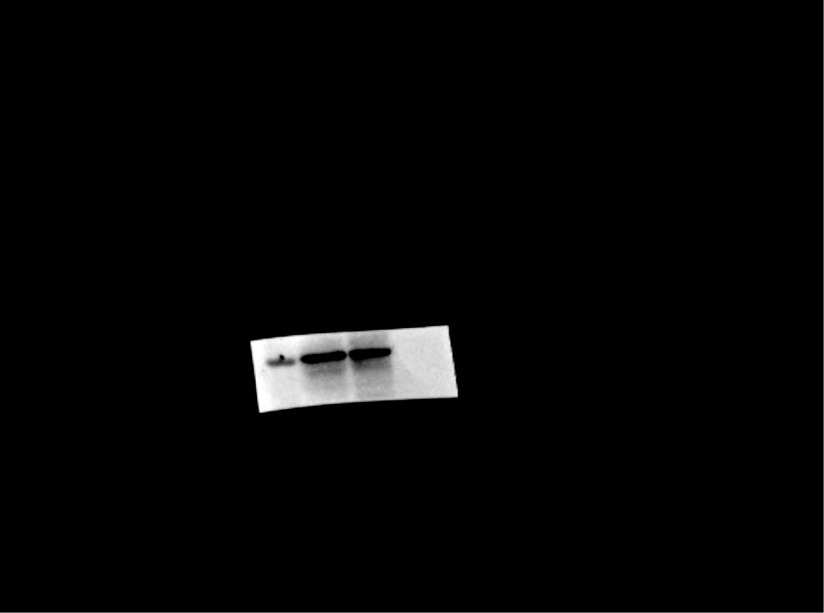


Fig. 5E-4


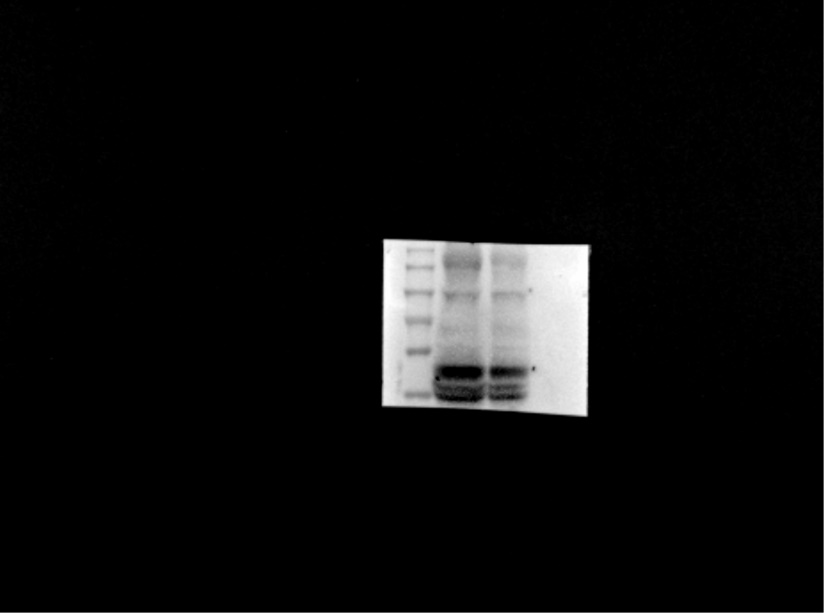


Fig. 5E-5


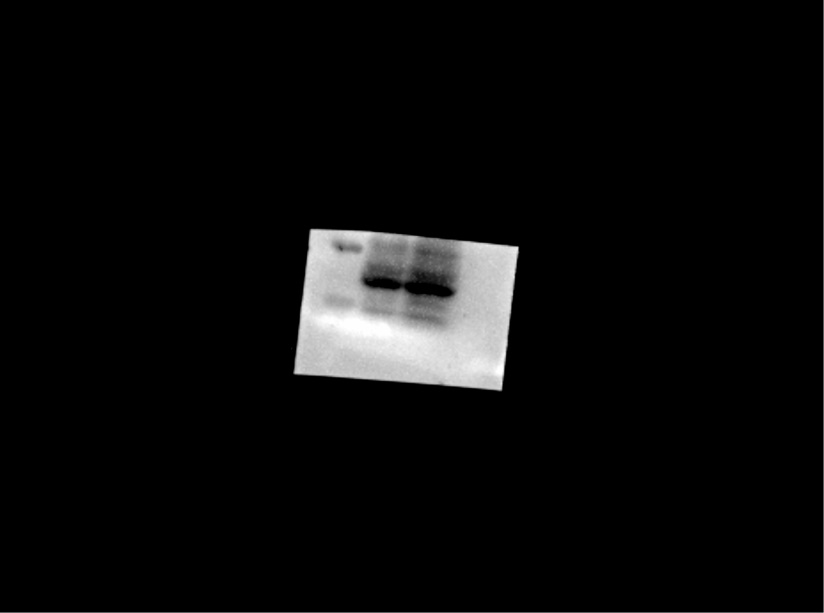


Fig. 5E-6


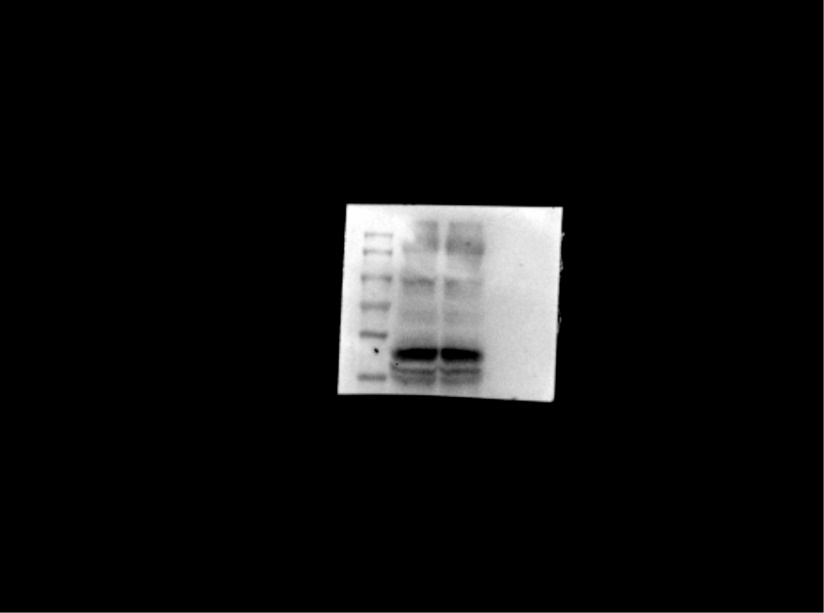


Fig. 6E-1


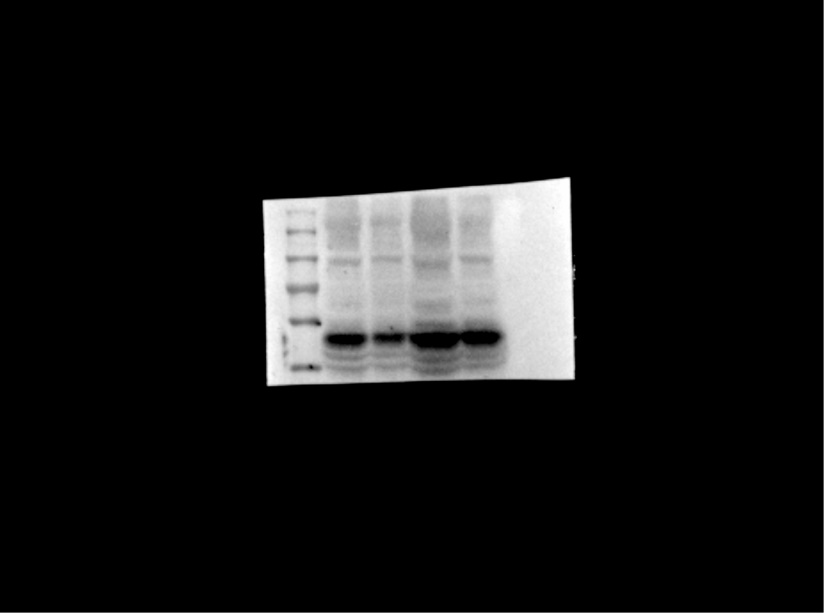


Fig. 6E-2


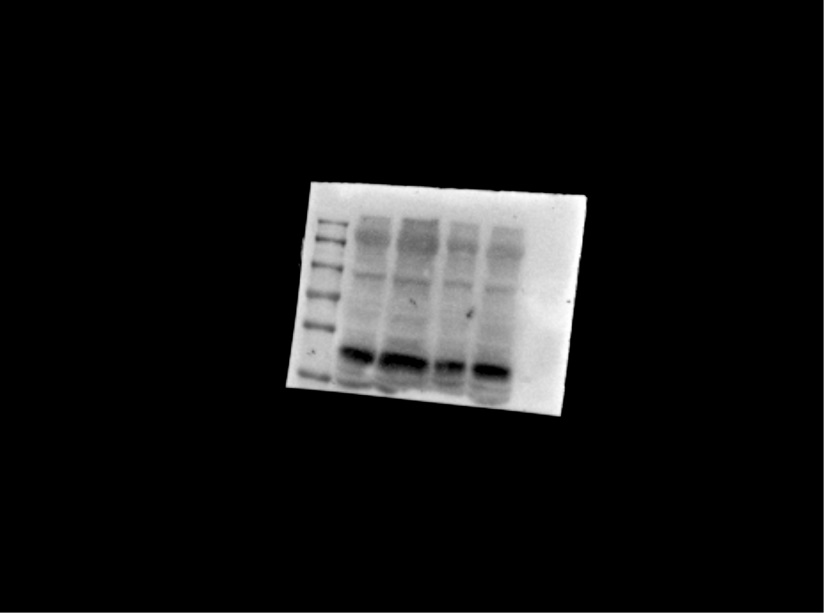


Fig. 6E-3


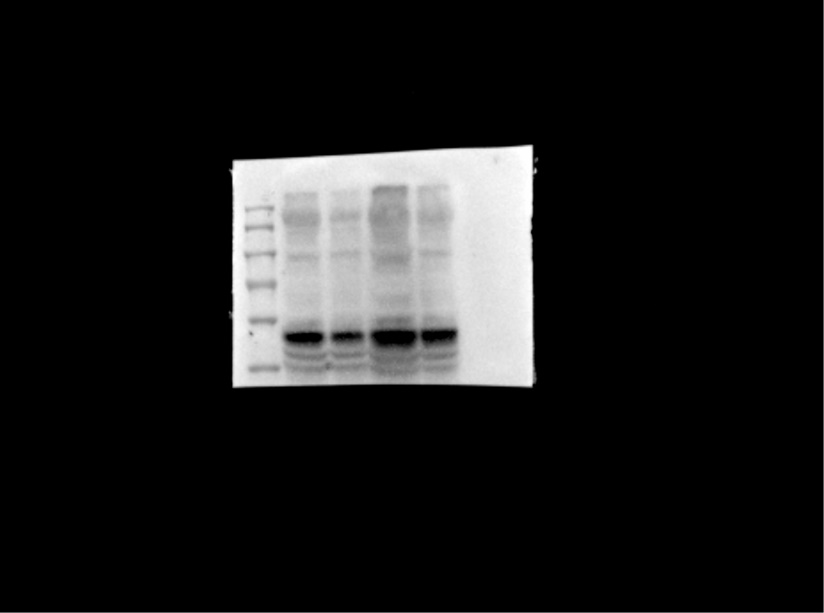


Fig. 6E-4


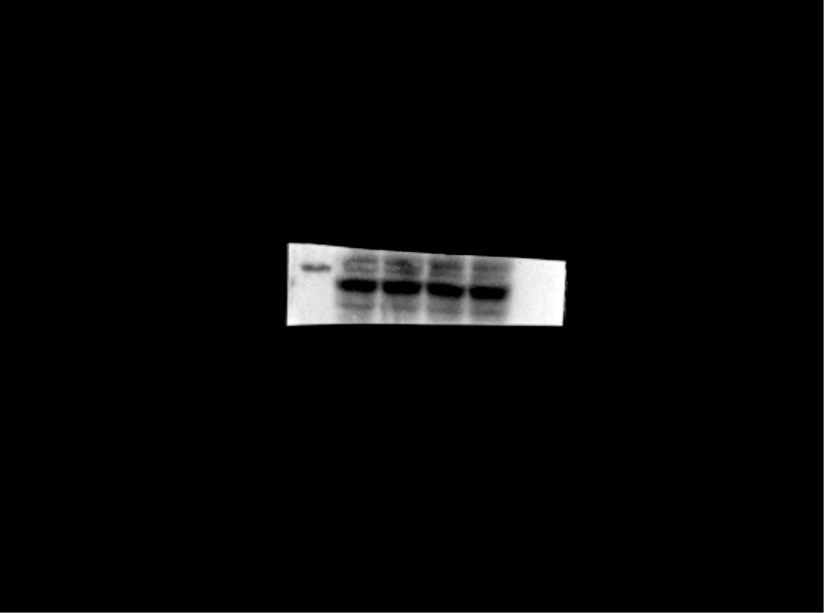


Fig. 7B-1


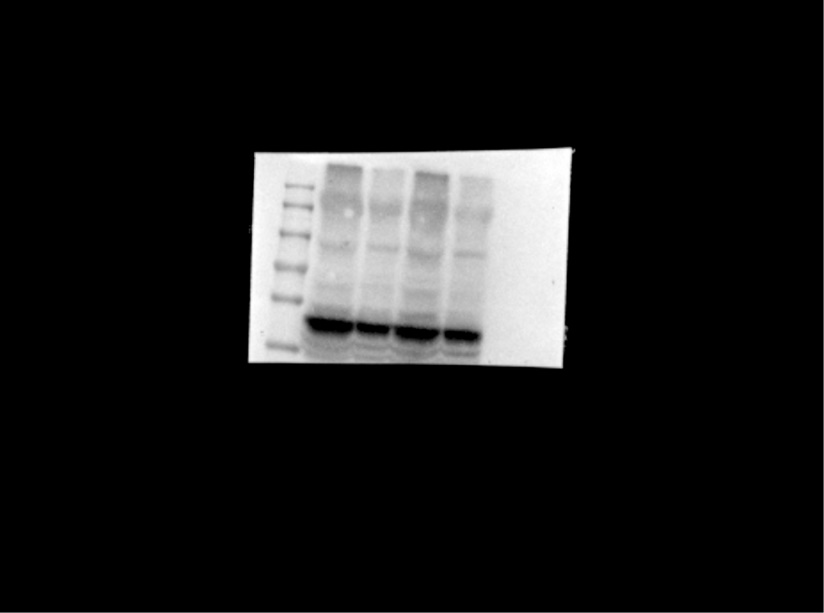


Fig. 7B-2


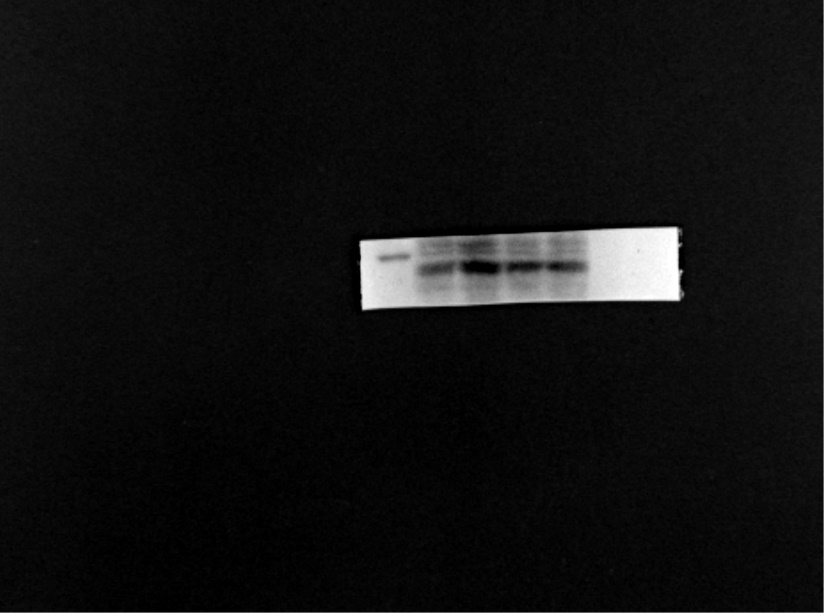


Fig. 7B-3


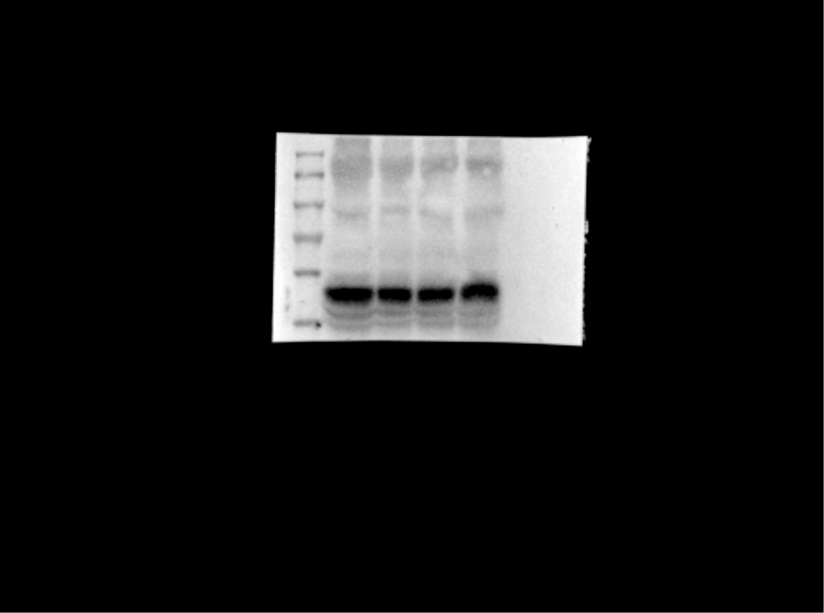


Fig. 7B-4


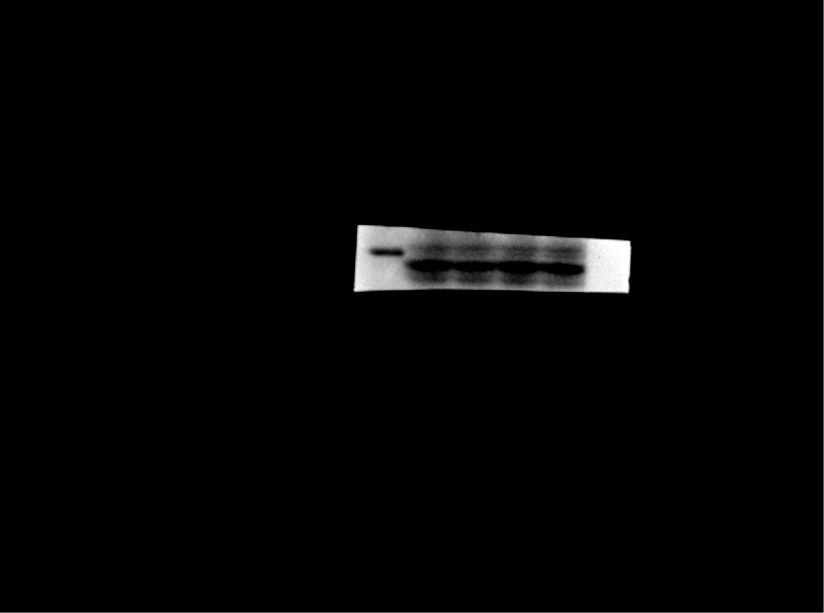

Supplement: Supplementary file 3 — WB Figures [file 41419_2023_5795_MOESM3_ESM.docx]
